# JAK-Inhibitors Beyond the Label: Emerging Applications in Dermatology

**DOI:** 10.3390/medicina62010190

**Published:** 2026-01-16

**Authors:** Giulio Foggi, Francesco D’Oria, Costanza Falcidia, Matteo Bianco, Luciano Ibba, Alessandra Narcisi, Antonio Costanzo, Luigi Gargiulo

**Affiliations:** 1 Dermatology Unit, IRCCS Humanitas Research Hospital, 20089 Rozzano, Italy; giulio.foggi@humanitas.it (G.F.); francesco.doria@humanitas.it (F.D.); costanza.falcidia@humanitas.it (C.F.); matteo.bianco@humanitas.it (M.B.); luciano.ibba@humanitas.it (L.I.); alessandra.narcisi@humanitas.it (A.N.); antonio.costanzo@hunimed.eu (A.C.); 2 Department of Biomedical Sciences, Humanitas University, 20072 Pieve Emanuele, Italy

**Keywords:** abrocitinib, baricitinib, JAK-inhibitors, ritlecitinib, upadacitinib

## Abstract

*Background and Objectives:* Oral Janus kinase (JAK) inhibitors have become an important therapeutic class in dermatology, with approved indications including atopic dermatitis and alopecia areata. Owing to their broad immunomodulatory effects and rapid onset of action, these agents are increasingly used off label for a variety of inflammatory skin disorders that are often refractory to standard therapies. The objective of this review was to provide a comprehensive overview of the published literature on the off-label dermatologic use of oral JAK inhibitors, summarizing clinical outcomes, safety profiles and treatment durations reported in real-world settings. *Materials and Methods:* A literature search was conducted in PubMed to identify case reports and case series describing off-label dermatologic use of baricitinib, abrocitinib, upadacitinib, and ritlecitinib. Extracted data included authorship and year, article type, treatment regimen, treatment duration and follow-up, prior systemic therapies, clinical outcomes, and reported adverse events. *Results:* A total of 136 articles were included, comprising 45 articles on abrocitinib (63 patients), 55 on upadacitinib (94 patients), 35 on baricitinib (45 patients), and 2 on ritlecitinib (2 patients). Across a wide spectrum of dermatological conditions, oral JAK inhibitors showed consistent clinical efficacy. Responses were frequently rapid and disease control was often maintained over several months of treatment. In many cases, dose reduction or treatment discontinuation did not lead to immediate relapse. Overall tolerability was favorable, with adverse events reported in a minority of patients and predominantly described as mild and transient. *Conclusions:* Although our data is limited to case-based literature, this review highlights the broad off-label therapeutic potential of oral JAK inhibitors in dermatology. Their rapid onset of action, sustained clinical responses, frequent maintenance of remission after dose tapering or discontinuation and generally acceptable safety profile support their consideration as treatment options in selected patients.

## 1. Introduction

**Figure 1:**
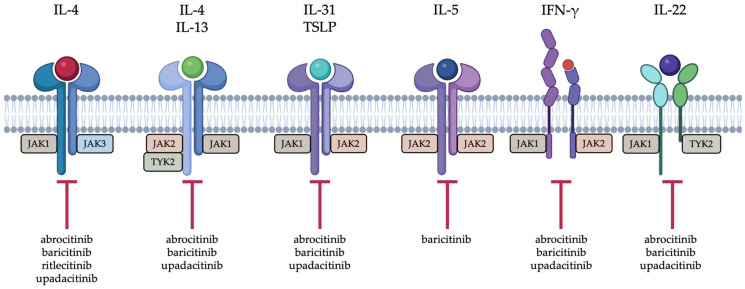
Schematic representation of cytokine receptor signaling pathways modulated by JAK inhibitors. The figure illustrates the main interleukins (IL-4, IL-13, IL-31, TSLP, IL-5, IFN-γ, and IL-22) and their associated receptor complexes, highlighting the involvement of specific kinases (e.g., JAK1, JAK2, JAK3, TYK2). The inhibitory actions of abrocitinib, baricitinib, ritlecitinib, and upadacitinib are indicated below each pathway.

Janus kinase (JAK) inhibitors have rapidly become an important therapeutic class in dermatology, reshaping the management of several chronic inflammatory and autoimmune skin diseases. Among the orally administered agents, upadacitinib, abrocitinib, baricitinib, and ritlecitinib are now established treatment options for moderate-to-severe atopic dermatitis and severe alopecia areata. Their clinical utility derives from the ability to modulate multiple cytokine pathways simultaneously, interrupting the Janus kinase—signal transducer and activator of transcription (JAK-STAT) signaling (Figure 1) [1,2,3,4,5,6,7,8,9,10,11].

Baricitinib is approved by both the Food and Drug Administration (FDA) and the European Medicines Agency (EMA) for the treatment of severe alopecia areata in adults [1,2]. Baricitinib is also approved in several regions, including the European Union (EU) and United Kingdom (UK), for moderate-to-severe atopic dermatitis in adults who are candidates for systemic therapy and have an inadequate response or contraindication to topical or conventional systemic treatments [3,4]. Baricitinib, a selective and reversible Janus kinase 1 (JAK1) and Janus kinase 2 (JAK2) inhibitor, blocks signaling of many cytokines such as intereukin-6 (IL-6), interleukin-12 (IL-12), interleukin-23 (IL-23), interferons, and granulocyte-macrophage colony-stimulating factor (GM-CSF). Through this broader inhibition, it reduces dendritic-cell activation, B-cell differentiation, and T helper 1 (Th1)/T helper 17 (Th17)-driven T-cell responses, mechanisms relevant to inflammatory dermatoses including alopecia areata and atopic dermatitis [1,2,3,4,12].

Abrocitinib is approved by both the EMA and FDA for moderate-to-severe atopic dermatitis. In the United States, it is approved for adults ≥ 18 years who have failed or are intolerant to systemic therapies, whereas the EMA authorizes its use in patients ≥ 12 years who are candidates for systemic treatment [5,6,7]. Abrocitinib is a highly selective JAK1 inhibitor that interferes with interleukin-4 (IL-4), interleukin-13 (IL-13), interleukin-22 (IL-22), interleukin-31 (IL-31), and thymic stromal lymphopoietin (TSLP) signaling, key drivers of type-2 inflammation and pruritus. Its sparing of JAK2 contributes to a more favorable hematologic profile [10,13,14].

Upadacitinib is approved for moderate-to-severe atopic dermatitis in adults and adolescents ≥ 12 years whose disease is inadequately controlled with systemic therapies or for whom such treatments are inappropriate [10,11]. Upadacitinib, also a selective JAK1 inhibitor, disrupts a similar range of JAK1-dependent cytokines, including IL-6, IL-4, IL-13, IL-22, IL-31 and various interferons. Its strong preference for JAK1 over JAK2, Janus kinase 3 (JAK3), and tyrosine kinase 2 (TYK2) helps limit off-target hematopoietic effects and underlies its efficacy in diseases where JAK1-mediated pathways dominate [15].

Finally, ritlecitinib, is approved by the FDA and EMA for severe alopecia areata in patients ≥ 12 years. Ritlecitinib distinguishes itself by its covalent, irreversible inhibition of JAK3 and tyrosine kinase expressed in hepatocellular carcinoma (TEC) kinases, providing >10,000-fold specificity for JAK3 over other JAK isoforms. By blocking γ-chain cytokine signaling (e.g., IL-15–STAT5 activation) and TEC-mediated B-cell activation, ritlecitinib effectively suppresses pathogenic cytotoxic T cells and restores hair-follicle immune privilege in alopecia areata [8,9].

Despite differences in selectivity, all agents converge on downregulation of STAT-dependent transcription, thereby dampening inflammatory circuits and contributing to clinical improvement across a broad spectrum of dermatologic conditions. For this reason, beyond these approved uses, oral JAK inhibitors are increasingly being prescribed off-label for a wide range of refractory dermatoses, with promising results [16].

This article aims to provide a comprehensive overview of the off-label dermatologic uses of oral JAK inhibitors, drawing from the growing body of case reports and case series published since these agents entered clinical practice. By reviewing the available literature, we seek to highlight their emerging efficacy across a wide spectrum of skin diseases for which no standardized JAK-based therapy currently exists. This review also summarizes reported therapeutic combinations, identifying scenarios in which JAK inhibitors have been used synergistically with other systemic agents, as well as documenting adverse events, tolerability considerations and the treatment durations required to achieve meaningful clinical responses. In doing so, we aim to offer clinicians a clearer understanding of how these agents are being applied beyond their approved indications and the potential role they may play in managing complex or refractory dermatologic conditions.

## 2. Materials and Methods

A literature search was conducted to identify case reports and case series describing the off-label dermatologic use of oral JAK inhibitors, specifically baricitinib, abrocitinib, upadacitinib, and ritlecitinib. Only publications reporting applications of these agents in dermatologic conditions outside their approved indications were included. Ongoing clinical trials and studies evaluating approved uses were excluded. Literature search was conducted between 1 October 2025 and 30 November 2025. All relevant articles published before October 2025 were included.

The search was performed in PubMed, using the following queries for each drug:

- *“abrocitinib” AND “dermatology”*
- *“upadacitinib” AND “dermatology”NO “atopic”*
- *“baricitinib” AND “dermatology”NO “atopic” NO “alopecia”*
- *“ritlecitinib” AND “dermatology”*

The “Case Reports” filter was applied in all searches to restrict results to individual case descriptions and small case series. Given the recent introduction of ritlecitinib into clinical practice, articles categorized as “Letters” were also considered for this molecule to capture all relevant early clinical experiences.

For each included report, we compiled a structured table organized by individual drug, documenting the following: first author and year of publication, disease, article type, therapeutic regimen, treatment duration and follow-up, prior systemic therapies, clinical outcomes, and any reported adverse events. This approach allowed for a qualitative synthesis of emerging real-world evidence regarding the off-label dermatologic applications of oral JAK inhibitors.

## 3. Results: Baricitinib

### 3.1. Current Indications

Baricitinib, an oral selective inhibitor of JAK 1 and JAK2, has emerged as a key systemic therapy in dermatology, with regulatory approval for two major inflammatory skin diseases: moderate-to-severe atopic dermatitis and severe alopecia areata [2,17].

### 3.2. Off-Label Use

Baricitinib is increasingly recognized not only for its approved indications in moderate-to-severe atopic dermatitis and severe alopecia areata, but also for a rapidly expanding spectrum of off-label applications in dermatology [18]. A growing body of case reports and small series highlights its potential utility across diverse inflammatory and immune-mediated skin diseases. These include autoimmune bullous diseases, such as mucous membrane pemphigoid, epidermolysis bullosa pruriginosa, and bullous pemphigoid, including drug-induced and infantile variants [19,20,21,22,23,24,25], as well as connective tissue and lupus-related dermatoses, encompassing subacute cutaneous lupus erythematosus, juvenile and blaschko-linear lupus, cutaneous polyarteritis nodosa, and linear morphea [26,27,28,29,30]. Evidence also supports efficacy in lichenoid and sclerosing disorders such as frontal fibrosing alopecia, nail lichen planus, and lichen sclerosus [31,32,33,34], and in neutrophilic or autoinflammatory conditions like pyoderma gangrenosum, interferon-stimulated gene 15 (ISG15) deficiency, chronic atypical neutrophilic dermatosis with lipodystrophy and elevated temperature (CANDLE) syndrome, Sweet syndrome, and synovitis, acne, pustulosis, hyperostosis, and osteitis (SAPHO) syndrome [35,36,37,38,39,40].

Additional reports document benefits in follicular and hair-follicle-associated dermatoses, such as eosinophilic pustular folliculitis and dissecting cellulitis of the scalp [41,42], reactive dermatoses and photodermatoses including actinic prurigo, chronic pruritus, and eruptive pruritic papular porokeratosis [43,44,45,46,47], and in fibrosing or metabolic disorders such as granuloma annulare, Morbihan disease, and reactive perforating collagenosis [48,49,50]. Finally, promising repigmentation outcomes have been observed in pigmentary and autoimmune melanocytic disorders like vitiligo [51,52,53].

The underlying rationale for these exploratory uses lies in baricitinib’s capacity to inhibit the JAK–STAT signaling pathway, thereby modulating multiple cytokines implicated in cutaneous autoimmunity and inflammation. In the following section, we summarize the available evidence on these emerging off-label indications, highlighting clinical outcomes, safety findings, and the mechanistic rationale supporting their investigation. A more detailed overview of the data is provided in the table below (Table 1).

#### 3.2.1. Autoimmune Bullous Diseases

Growing evidence supports the potential role of JAK inhibition, particularly baricitinib, in the management of autoimmune subepidermal blistering diseases traditionally refractory to standard immunosuppression. Reports include its successful use in mucous membrane pemphigoid (MMP) with multisystem involvement unresponsive to corticosteroids, intravenous immunoglobulin IVIG, and cyclophosphamide, where the combination of baricitinib and methotrexate achieved sustained mucosal and ocular remission with restoration of oral intake [19].

In epidermolysis bullosa pruriginosa, oral baricitinib 2 mg daily led to remarkable clinical improvement, with a marked reduction in pruritus and flattening of nodular lesions without adverse effects [20].

Similarly, several reports describe efficacy in bullous pemphigoid (BP), including both classic and variant forms. In an elderly patient with uncontrolled diabetes, baricitinib induced complete remission within ten weeks while avoiding systemic steroid complications [21]. Another case of BP associated with plaque psoriasis achieved simultaneous remission of both diseases with baricitinib 4 mg daily, supporting its dual anti-inflammatory action across T helper 2 (Th2)- and Th17-mediated pathways [23]. In pediatric populations, even infantile refractory BP resistant to corticosteroids, IVIG, and dupilumab responded completely to baricitinib, with full remission and no relapse after discontinuation [25].

Furthermore, baricitinib proved effective in lichen planus pemphigoides, achieving near-complete resolution after six months of therapy in a long-standing, treatment-refractory case [22]. Lastly, in a complex case of drug-induced BP and lupus erythematosus secondary to anti-tumor necrosis factor (anti-TNF) and IL-6 blockade in rheumatoid arthritis, the introduction of baricitinib resulted in disease control without recurrence of blistering or hematologic toxicity [24].

Collectively, these observations demonstrate consistent improvement across the spectrum of autoimmune bullous diseases, rapid resolution of inflammation and pruritus, and sustained remission even in recalcitrant or steroid-contraindicated cases.

#### 3.2.2. Cutaneous Lupus and Connective Tissue Dermatoses

In cutaneous lupus and connective-tissue dermatoses, baricitinib shows converging benefits across interferon-driven inflammation and fibrosing pathways. In subacute cutaneous lupus erythematosus (SCLE) with concomitant frontal fibrosing alopecia, short-course baricitinib induced complete SCLE clearance and halted further alopecia progression, highlighting JAK-STAT blockade as a unifying target for both photosensitive lupus plaques and lichenoid-scarring alopecia [26]. In juvenile SLE with a pathogenic three prime repair exonuclease 1 (TREX1) variant and a strong type-I interferon signature, adding baricitinib to mycophenolate rapidly improved severe, refractory cutaneous vasculitis and enabled steroid minimization, supporting a precision-medicine rationale where genotypic/interferon (IFN) signature enrichment predicts JAK-inhibitor responsiveness [27]. For rarer cutaneous lupus erythematosus (CLE) phenotypes, blaschko-linear lupus flattened with 4 mg/day baricitinib and maintained stability months after discontinuation, suggesting durable disease control once the inflammatory cascade is reset [28]. Beyond lupus, baricitinib also improved medium-vessel cutaneous vasculitis (cutaneous polyarteritis nodosa) with ulcer healing, livedo reduction, and steroid tapering—useful where conventional agents fail or are poorly tolerated [29]. Finally, in linear morphea along the lines of Blaschko, a year of low-dose therapy softened sclerosis and improved mobility; immunohistochemistry showed increased IL-4 and transforming growth factor beta-1 (TGF-β1) expression, and the known downstream JAK signaling provides a mechanistic bridge between antifibrotic and anti-inflammatory effects [30]. Taken together, these reports suggest baricitinib can act as a steroid-sparing option across CLE spectrum and selected connective-tissue vasculitides/sclerosing disorders, with quicker pruritus/erythema control in interferon-high phenotypes and progressive softening in fibrosing disease, while early signals of tolerability are favorable across adult and pediatric cases [26,27,28,29,30].

#### 3.2.3. Lichenoid and Sclerosing Dermatoses

Baricitinib has shown promising and broad efficacy across the spectrum of lichenoid and sclerosing dermatoses, targeting both interface dermatitis and fibrosing immune pathways. In lichen planopilaris (LPP) and its frontal fibrosing variant (FFA), baricitinib offers meaningful disease control in patients refractory to conventional therapies such as corticosteroids, antimalarials, and 5α-reductase inhibitors. In a multicenter case series, baricitinib and other JAK inhibitors led to significant reductions in perifollicular erythema, scale, and pruritus, with partial hair regrowth in some cases, supporting the concept that JAK-STAT blockade may restore follicular immune privilege and interrupt interferon-driven cytotoxic injury [31]. The results mirror those previously observed in smaller cohorts using oral and topical JAK inhibitors, suggesting a potential role for early integration of baricitinib in LPP/FFA management, particularly where scarring progression threatens irreversible alopecia.

In isolated nail lichen planus (NLP), where traditional therapies frequently fail and functional impairment can be severe, baricitinib 4 mg daily achieved complete nail recovery within six months and maintained remission over a year, without adverse effects [32]. Mechanistically, this aligns with data demonstrating that baricitinib blocks interferon-gamma (IFN-γ)-driven CD8^+^ cytotoxicity against keratinocytes, a hallmark of lichenoid pathology.

The JAK1/2 inhibition strategy extends to lichen sclerosus (LS), a chronic sclerosing dermatosis characterized by Th1 cytokine predominance and tissue fibrosis. Both pediatric and adult cases, including those triggered by COVID-19 vaccination, showed rapid clinical and dermoscopic improvement after oral baricitinib, often within weeks, with marked softening and repigmentation of lesions [33,34]. Notably, baricitinib was effective as monotherapy in extragenital LS and well tolerated even in a two-year-old child, underscoring its immunomodulatory versatility.

Across these reports, a common thread emerges: baricitinib mitigates lichenoid interface inflammation and downstream fibrosing remodeling by dampening interferon and T-cell-mediated pathways. The comparative responses in inflammatory (LPP, NLP) versus fibrosing (LS) entities suggest that JAK inhibition exerts both anti-inflammatory and antifibrotic effects, bridging mechanisms that link the lichenoid–sclerosing disease spectrum [31,32,33,34].

#### 3.2.4. Neutrophilic and Autoinflammatory Skin Disorders

Across neutrophilic and autoinflammatory skin disorders, baricitinib consistently shows benefit, often after failure of conventional or biologic therapies, and its fastest responses appear in entities with strong interferon signaling. In pyoderma gangrenosum (PG), baricitinib has been associated with complete ulcer healing in reports and meaningful improvement within a 12-week course alongside steroid tapering, underscoring utility in a classic neutrophilic dermatosis with limited high-level evidence for any single therapy [35]. In interferon-driven diseases, effects can be striking: ISG15 deficiency with severe ulcerative skin disease showed complete and rapid resolution on baricitinib, highlighting JAK blockade as a pathogenesis-directed treatment for type-I/II interferonopathies, while CANDLE/proteasome-associated autoinflammatory syndrome (PRAAS) likewise achieved normalization of cutaneous and systemic inflammation on baricitinib, supporting a shared mechanism of benefit via dampening type I interferon signaling [36,37]. Among neutrophilic dermatoses linked to rheumatic disease, baricitinib rapidly controlled rheumatoid arthritis (RA)-associated Sweet syndrome with remission of both skin and joint activity by 4 weeks, suggesting dual dermatologic–rheumatologic efficacy where IL-6/IFN-γ-related pathways are implicated [38]. For SAPHO syndrome, a heterogeneous autoinflammatory condition with neutrophil-rich lesions, baricitinib improved both pustular/psoriasiform skin disease and osteoarticular pain within two weeks, positioning JAK inhibition as a potential option when nonsteroidal anti-inflammatory drugs (NSAIDs), disease-modifying antirheumatic drugs (DMARDs), and TNF inhibitors underperform [39]. Notably, baricitinib has also rescued paradoxical neutrophilic/eczematous reactions (PG and eczema) that emerged during biologic therapy for psoriasis, reinforcing its role as a versatile, pathway-targeted agent when cytokine-specific biologics trigger off-target inflammation [40]. Taken together, across PG, Sweet syndrome, SAPHO, and interferonopathies (ISG15 deficiency, CANDLE), the recurring pattern is rapid cutaneous improvement, particularly when disease biology is interferon-skewed.

#### 3.2.5. Follicular and Hair-Follicle-Associated Dermatoses

Across follicular and hair-follicle-associated dermatoses, baricitinib shows promise both as monotherapy and as an adjunct when follicular occlusion disease proves refractory. In eosinophilic pustular folliculitis (EPF) resistant to long-term indomethacin, daily baricitinib 4 mg produced a rapid clinical response within 5 days with normalization of eosinophils/IgE and successful dose-tapering without adverse events, supporting a type-2 cytokine/JAK-STAT-driven mechanism in EPF and positioning baricitinib as a steroid-sparing option [41]. By contrast, in perifolliculitis capitis abscedens et suffodiens (PCAS, dissecting cellulitis), where neutrophilic scarring alopecia and sinus tracts often persist despite antibiotics and isotretinoin, adding baricitinib (4 mg) to adalimumab after suboptimal response led to near-complete lesion control and visible hair regrowth over 9 months, suggesting complementary benefits of JAK inhibition alongside TNF-α blockade in the follicular occlusion spectrum and hinting at a role for baricitinib when lipid toxicity or plateauing efficacy limits isotretinoin or biologic monotherapy [42]. Considered together, these reports highlight faster symptom relief and biomarker improvements in EPF with baricitinib alone versus structural recovery and regrowth in PCAS when baricitinib is layered onto TNF inhibition, an association consistent with differing inflammatory milieus (Th2-skewed eosinophilic vs. neutrophilic occlusive disease) yet a shared reliance on JAK-mediated signaling across the follicular disorders spectrum [41,42].

#### 3.2.6. Photodermatoses and Reactive Pruritic Dermatoses

Across photodermatoses and reactive pruritic dermatoses, baricitinib shows rapid antipruritic effects and durable lesion control, with signals of efficacy across distinct immune phenotypes. In chronic pruritus of unknown origin refractory to multiple modalities, including dupilumab, oral baricitinib induced near-complete itch relief within 5 days and sustained remission after brief exposure, underscoring a neurosensory/Type-2-linked itch pathway amenable to JAK blockade [44]. In actinic prurigo, baricitinib 4 mg/day drove marked pruritus reduction from week 1 and complete clinical clearing by 4 months in an adult with 18-month relapse-free follow-up, while a pediatric case achieved clearance of cheilitis and photo-exposed papules within 2 weeks on 2 mg/day and remained asymptomatic at 12 weeks, together suggesting that down-regulation of intertwined Th1/Th2 cytokine signaling can rapidly reverse both symptoms and lesions [45,46]. Extending beyond classic photodermatoses, severe ulcerative lipodystrophia centrifugalis abdominalis infantilis improved with hydroxychloroquine and then fully healed after adding low-dose baricitinib, with stability after drug withdrawal, highlighting complementary use (combination vs. monotherapy) and a possible role for JAK inhibition in panniculitic inflammation where standard agents only partially succeed [47]. Collectively, these reports associate baricitinib with faster itch relief than many comparators, complete lesion resolution in actinic prurigo, and utility as an adjunct in complex ulcerative disease, patterns consistent with broad suppression of pruritogenic and photo-provoked cytokine circuits via JAK-STAT modulation [44,45,46,47].

#### 3.2.7. Reactive, Metabolic, and Fibrosing Dermatologic Disorders

Across reactive, metabolic, and fibrosing dermatologic disorders, baricitinib demonstrates rapid antipruritic and anti-inflammatory activity with disease-specific response kinetics and some relapse on withdrawal. In generalized granuloma annulare, two refractory cases cleared dramatically within 4–6 weeks on 4 mg/day, with recurrence after self-discontinuation in one patient, highlighting both speed of effect and a potential need for maintenance [49]. In acquired reactive perforating collagenosis, a pruritic disorder frequently linked to diabetes, 2 mg/day induced progressive improvement culminating in complete lesion and itch resolution by week 8; symptoms recurred after stopping and remitted again on re-initiation, aligning with emerging evidence of Th2-skewed pruritic inflammation responsive to JAK blockade [50]. In Morbihan disease—a chronic fibrosing/edematous facial condition refractory to multiple prior therapies—daily 2 mg produced marked reduction in erythema and edema within 4 weeks, representing the first reported use of baricitinib in this entity and underscoring JAK inhibition as a plausible, mechanism-bridging option when lymphatic dysfunction and inflammation intersect [48]. Considering these together, baricitinib’s fastest clearance appears in reactive granulomatous disease (GA), intermediate in fibrosing facial edema (Morbihan), and somewhat slower in metabolic-associated perforating disease (ARPC); durability often improves with continued dosing, and mechanistic convergence on JAK-mediated cytokine circuits (Th1 in GA, Th2/pruritogenic in ARPC, mixed inflammatory/vascular-lymphatic in Morbihan) helps explain the broad yet coherent efficacy profile [48,49,50].

#### 3.2.8. Pigmentary and Autoimmune Melanocytic Disorders

Across pigmentary and autoimmune melanocytic disorders, principally vitiligo, baricitinib has shown reproducible repigmentation, especially when paired with controlled light exposure. Case reports and series describe meaningful pigment return within months: two generalized vitiligo patients achieved early repigmentation after 1 month and substantial clearing by 6–8 months on oral baricitinib combined with narrowband ultraviolet B (NB-UVB), aligning with IFN-γ/JAK-STAT suppression as a driver of response [51]. A comparative single-patient experience underscores potential superiority of JAK1/2 targeting: vitiligo failed to improve on tofacitinib (JAK1/3) but nearly completely repigmented after switching to baricitinib 4 mg daily for 8 months, suggesting mechanistic advantage when IFN-γ–JAK1/2 signaling predominates [52]. A five-patient series extends this synergy to real-world heliotherapy (moderate daily sun), reporting an average 25.9% reduction in Vitiligo Extent Score (VES)-estimated body surface area over 5 months, with best gains on photo-exposed sites; safety signals were mostly mild to moderate and manageable (e.g., transient creatine kinase rise, lipid changes, renal function dip prompting dose reduction), reinforcing the need for lab monitoring [53]. Taken together, these data support a pragmatic algorithm: leverage baricitinib to mitigate IFN-γ-driven autoimmunity and pair it with controlled UV/light to stimulate melanocyte activity—an association that appears to accelerate and broaden repigmentation compared with JAK inhibition alone, and that may outperform JAK1/3 blockade in select refractory cases [51,52,53].

## 4. Results: Abrocitinib

### 4.1. Current Indications

Abrocitinib is a small-molecule Janus kinase inhibitor that selectively targets JAK1. It is approved by both the EMA and the FDA for the treatment of moderate-to-severe atopic dermatitis, administered at a daily dose of 100 or 200 mg. The FDA has approved its use in adults aged ≥ 18 years who have failed or are intolerant to systemic therapy, whereas the EMA has authorized its use in patients aged ≥ 12 years who are candidates for systemic treatment [5,6,7,54,55,56]. Real-world evidence has largely confirmed the safety profile observed in clinical trials while also supporting the effectiveness of abrocitinib in routine clinical practice for atopic dermatitis [57].

### 4.2. Off-Label Use

Although abrocitinib is a selective JAK1 inhibitor, its targeted mechanism still enables modulation of multiple signaling pathways implicated in the pathogenesis of a broad range of inflammatory and immune-mediated diseases [13].

In this section, we review published case reports and case series describing the off-label use of abrocitinib beyond atopic dermatitis. The available literature documents its successful use, albeit sometimes partial, in a wide variety of dermatologic conditions, including bullous diseases [58], connective tissue disorders [59], lichen planus [60,61,62,63], hydradenitis suppurativa [59], neutrophilic dermatoses [59,64,65,66], scalp disorders [67,68,69,70], and dermatitis due to exogenous causes [71,72].

Additionally, published reports describe the use of abrocitinib in rosacea [73,74,75,76,77], granulomatous diseases [78,79,80,81,82,83], vitiligo [84,85,86], prurigo nodularis [87,88], chronic spontaneous urticaria [89], genodermatoses [90,91,92,93], lichen amyloidosis [94,95], as well as in a heterogeneous group of other inflammatory skin disorders [96,97,98,99].

A more detailed overview of the data is provided in the table below (Table 2).

#### 4.2.1. Bullous Diseases

Abrocitinib may represent a valuable therapeutic option for mucous membrane pemphigoid (MMP), particularly in patients who are unable or unwilling to receive systemic corticosteroids or immunosuppressive therapies. In this report, a 62-year-old woman with a six-month history of painful oral erosions and scattered cutaneous blisters, unresponsive to high-potency topical corticosteroids and tacrolimus, achieved rapid improvement after initiating abrocitinib 100 mg daily. Pain decreased significantly within three days, oral erosions began healing by the second week, and complete resolution was observed at four weeks, accompanied by drying of cutaneous blisters and normalization of anti-bullous pemphigoid antigen 180 (anti-BP180) titers. Treatment was well tolerated, allowing dose tapering and subsequent discontinuation without relapse during follow-up. The JAK–STAT pathway participates in the immunopathogenesis of bullous pemphigoid and related disorders. By inhibiting JAK1-dependent signaling for cytokines such as IFN-γ and IL-4, abrocitinib may reduce autoantibody-driven inflammation and modulate Th2-skewed responses implicated in loss of tolerance to BP180, offering a plausible explanation for the rapid symptomatic improvement seen in this case [58].

#### 4.2.2. Connective Tissue Dermatoses

In this off-label setting, a 31-year-old woman with livedoid vasculopathy, presenting with a more than two-year history of recurrent painful papules progressing to ulceration and necrosis and refractory to multiple conventional systemic therapies, was treated with oral abrocitinib 100 mg daily. The off-label JAK1 inhibition resulted in rapid symptomatic improvement within one week and complete ulcer remission by week six, with only post-inflammatory hyperpigmentation persisting and no adverse events or recurrence reported throughout a 12-week follow-up. Although this is primarily a thrombo-occlusive disorder, inflammatory cytokines, particularly type I interferons and IL-6 family mediators, contribute to endothelial injury and microvascular thrombosis. By selectively inhibiting JAK1-dependent pathways, abrocitinib may reduce endothelial inflammation and downstream coagulation-driven damage, providing a plausible explanation for the rapid ulcer healing observed in this case [59].

#### 4.2.3. Lichen Planus

Abrocitinib, a selective JAK1 inhibitor approved for atopic dermatitis, is increasingly being explored off label in the management of lichen planus, with accumulating evidence supporting its potential across multiple phenotypes. The most compelling data come from nail lichen planus (NLP), a variant known for its therapeutic refractoriness and risk of irreversible nail damage [60,61,62,63].

In a first case of isolated NLP, a 39-year-old woman with progressive longitudinal ridging, onycholysis and subungual hyperkeratosis experienced pronounced improvement on abrocitinib 100 mg daily, achieving nearly 80–82% reductions in both total Nail Lichen Planus Severity Index (tNLPSI) and Dermatology Life Quality Index (DLQI) scores over six months, with further recovery on alternate-day dosing and no adverse events [60]. Another report involving severe, generalized NLP in a 31-year-old woman, affecting all 20 nails and causing near-complete architectural distortion, demonstrated similarly robust outcomes: after failure of topical therapy and refusal of systemic immunosuppression, treatment with abrocitinib 100 mg daily led to partial normalization at four months and substantial regrowth of healthy nail plates by seven months. A rapid flare after patient-initiated discontinuation further underscored the drug’s role in maintaining disease control, as re-treatment again reversed the worsening [61]. Collectively, these cases highlight the capacity of JAK1 inhibition to modulate the intense inflammatory activity underlying NLP, offering a therapeutic avenue where conventional therapies often fall short [60,61].

Beyond the nail apparatus, abrocitinib has also shown encouraging off-label outcomes in mucosal and genital forms of lichen planus. A 50-year-old woman with long-standing erosive and hypertrophic vulvar lichen planus refractory to an extensive list of systemic and topical therapies achieved early symptomatic relief within weeks of starting abrocitinib 200 mg daily, followed by progressive mucosal healing and approximately 95% remission at 20 months, accompanied by marked improvements in pain, sleep, and quality of life [62]. Similarly, in plasma cell balanitis associated with male genital lichen sclerosus, a 50-year-old man unresponsive to calcineurin inhibitors, corticosteroids, antiseptics, circumcision, and antimicrobial therapy achieved visible improvement within three days of initiating abrocitinib 100 mg daily and complete remission within one month, maintaining stable laboratory parameters throughout six months of follow-up [63].

Across these LP subtypes, JAK1 signaling plays a central pathogenic role, particularly through interferon-γ-driven pathways and downstream chemokines such as C-X-C motif chemokine ligand 10 (CXCL10). LP lesions show heightened activity of IFN-γ, interleuki-21 (IL-21), and Th1-skewed cytokines, many of which rely on JAK1 for intracellular signaling. By selectively inhibiting JAK1, abrocitinib attenuates this inflammatory cascade, offering a biologically coherent explanation for the rapid symptom relief and tissue healing observed across nail, vulvar, and mucosal disease variants [60,61,62,63].

#### 4.2.4. Hidradenitis Suppurativa

Abrocitinib may offer therapeutic benefit in hidradenitis suppurativa (HS), particularly in patients with chronic, treatment-refractory disease. In this report, a 17-year-old male with a three-year history of recurrent, painful axillary nodules and abscesses, unresponsive to systemic antibiotics, glucocorticoids, cyclosporine, methotrexate, and multiple surgical excisions, experienced rapid improvement after initiating abrocitinib 100 mg daily with short-term doxycycline. Within two weeks, pain and inflammatory swelling had markedly decreased. He subsequently maintained remission on alternate-day dosing, with no reported adverse events throughout follow-up. HS involves dysregulated Th1, Th17, and innate immune pathways with elevated cytokines, such as IL-6, interleukin-1 beta (IL-1β), IL-23, and type I interferons, that signal through JAK1. By blocking these upstream inflammatory circuits, abrocitinib may reduce follicular inflammation and neutrophil recruitment, providing a plausible explanation for the rapid and sustained clinical improvement observed [59].

#### 4.2.5. Neutrophilic Dermatoses

Abrocitinib, a selective JAK1 inhibitor approved for atopic dermatitis, is increasingly being explored off-label in neutrophilic dermatoses, conditions marked by intense cytokine-driven inflammation and frequent resistance to conventional therapies. In pyoderma gangrenosum (PG), for example, an adolescent with rapidly progressive perianal ulcerations refractory to multiple systemic treatments experienced clear clinical improvement within the first week of abrocitinib therapy, with steady ulcer reduction and excellent tolerability thereafter [59]. A similar rapid response was seen in an adult woman with a bullous variant of PG who had not improved despite hospitalization, antibiotics, and surgical debridement; pain, erythema, and ulcer activity decreased markedly soon after starting abrocitinib, ultimately leading to complete epithelialization during follow-up without adverse effects. These cases suggest that JAK1 inhibition may effectively modulate neutrophil-driven autoinflammation even in severe or treatment-resistant PG [64].

Abrocitinib has also shown potential utility in localized generalized pustular psoriasis (GPP). A middle-aged woman with pustular plaques confined to the lower legs—unresponsive to acitretin and misdiagnosed initially as eczema, experienced significant improvement and complete pustule resolution on abrocitinib 100 mg twice daily, later tapered to daily dosing. JAK1 signaling is involved in IL-2-induced upregulation of the interleukin-36 (IL-36) receptor, a key driver of GPP: by suppressing this axis, abrocitinib may limit IL-36-mediated neutrophilic inflammation [65].

Beyond classical neutrophilic dermatoses, abrocitinib has also shown benefit in autoinflammatory bone–skin diseases such as SAPHO syndrome. A teenager with severe acneiform eruptions and debilitating osteoarticular pain, minimally responsive to corticosteroids and methotrexate, achieved substantial symptomatic improvement shortly after starting abrocitinib. Cutaneous inflammation progressively subsided over the following months, joint pain completely resolved, and treatment was eventually discontinued without relapse. This case expands the therapeutic horizon of abrocitinib into syndromes where neutrophilic inflammation overlaps with osteitis and sacroiliac involvement [66].

Although clinically distinct, PG, GPP, and SAPHO share a core pathogenic feature: excessive neutrophil-driven inflammation mediated by JAK1-dependent cytokines, including IL-1 family members, IL-6, type I/II interferons, and neutrophil-recruiting chemokines. Abrocitinib’s selective JAK1 blockade likely dampens these upstream inflammatory circuits, reducing neutrophil activation and trafficking and thereby offering a coherent explanation for the rapid improvement observed across these three conditions [59,64,65,66].

#### 4.2.6. Hair and Scalp Diseases

Emerging case-based evidence indicates that abrocitinib may offer therapeutic benefit across several inflammatory scalp disorders, including alopecia universalis (AU) [67,68], alopecia areata (AA) [69], and dissecting cellulitis of the scalp (DCS) [70]. In AA, patients with long-standing or refractory disease, including adolescents and young adults with complete scalp and body hair loss, showed meaningful early regrowth after initiating abrocitinib. In one case, a 12-year-old boy with severe AD and mild AA unresponsive to dupilumab achieved complete hair regrowth within 12 weeks, with sustained remission over one year [69]. A 30-year-old woman who developed AU following drug reaction with eosinophilia and systemic symptoms (DRESS) exhibited initial regrowth on 100 mg of abrocitinib and more pronounced recovery after escalation to 200 mg, ultimately achieving substantial restoration of terminal scalp hair over six months [67]. Likewise, a 14-year-old girl with severe AD and a three-year history of vaccine-associated AU demonstrated progressive regrowth across the scalp, eyebrows, and limbs during the first year of treatment, with maintained disease control beyond two years [68].

Across these cases, treatment was well tolerated, and no significant laboratory abnormalities or adverse events were reported, even in pediatric patients and in individuals with complex immune backgrounds. Across these diseases, the therapeutic rationale for abrocitinib hinges on the central role of JAK1-dependent cytokine signaling in their shared immunopathology. AA is driven by cytotoxic CD8^+^ T cells activated through interferon-γ and interleukin-15 (IL-15), both of which signal via the JAK1–STAT pathway to sustain the autoimmune attack on hair follicles. By selectively inhibiting JAK1, abrocitinib dampens this upstream inflammatory circuit, reducing the IFN-γ/CXCL10 axis implicated in follicular destruction and thereby enabling hair regrowth [67,68,69].

Abrocitinib has also shown potential in DCS, a destructive follicular occlusion disorder marked by nodules, draining sinuses, and scarring. In a 27-year-old man with recalcitrant DCS unresponsive to antibiotics, retinoids, and biologics, abrocitinib 100 mg daily led to a marked reduction in inflammatory nodules and suppuration within four months; patient maintained remission for a full year on intermittent dosing without recurrence. DCS belongs to the follicular occlusion spectrum and is characterized by chronic neutrophil-driven inflammation. Evidence from the DCS report indicates involvement of JAK1-mediated cytokine pathways, including IL-6, IL-10, and IL-12/23, which sustain the persistent inflammatory reaction within hair follicles. By selectively inhibiting JAK1, abrocitinib may attenuate these pro-inflammatory signals, reducing neutrophil recruitment and follicular destruction [70].

Together, these cases highlight a recurring clinical pattern in scalp disorders treated off label with abrocitinib: early reduction in inflammatory burden, progressive follicular recovery, and durable disease stabilization, all achieved with a favorable safety profile. Although formal trials are lacking, the convergence of responses across AU, AA, and DCS supports further exploration of selective JAK1 inhibition as a therapeutic avenue for refractory scalp conditions driven by immune dysregulation [67,68,69,70].

#### 4.2.7. Reactive Exogenous Dermatitis

Emerging case evidence suggests that abrocitinib may offer therapeutic benefit in chronic inflammatory dermatoses beyond its established role in atopic dermatitis, including conditions such as chronic actinic dermatitis (CAD) [71] and perioral dermatitis (POD) [72].

In CAD, a disease characterized by photosensitive eczematous and lichenified plaques that often prove refractory to topical corticosteroids, immunomodulators, and photoprotection alone, treatment with abrocitinib 100 mg daily led to rapid improvement in pruritus, reported as early as the day after initiation, and substantial softening and regression of hypertrophic lesions over the subsequent weeks. Even in a patient with nearly two decades of persistent, symptomatic disease, abrocitinib produced marked symptomatic relief and visible clearing of sun-exposed plaques, with no adverse events observed during follow-up [71].

A similarly favorable response was reported in POD, a condition known for chronicity and frequent therapeutic recalcitrance. In a young woman with a one-year history of papulopustular lesions unresponsive to tetracyclines, calcineurin inhibitors, and elimination of corticosteroid-containing topical treatments, oral abrocitinib 100 mg daily resulted in rapid reduction in pruritus and near-complete resolution of lesions within two weeks, with remission maintained after discontinuation of therapy [72].

Both POD and CAD exhibit Th2-skewed inflammatory signaling, including cytokines such as IL-4, IL-13, IL-22, and IL-31, that rely on JAK1-dependent pathways. By inhibiting JAK1, abrocitinib reduces pruritus and downstream inflammatory amplification, offering a coherent explanation for the rapid symptom relief and lesion improvement observed across both conditions [71,72].

#### 4.2.8. Rosacea

Across rosacea-spectrum disorders and related facial edema, accumulating case-based data indicate that abrocitinib may provide clinically meaningful benefit in patients with disfiguring, treatment-refractory disease [73,74,75,76,77]. In solid facial edema (Morbihan syndrome), three men with long-standing rosacea-associated centrofacial swelling and erythema, unresponsive to antibiotics, isotretinoin, corticosteroids and other modalities, showed substantial and sustained reductions in edema, redness, and pruritus on abrocitinib 200 mg daily, with only mild residual eyelid changes in some cases and one transient grade I thrombocytopenia [73].

Several small series and case reports extend these observations to rosacea itself: four women with steroid-induced rosacea experienced rapid improvement in erythema, papules, burning, and quality-of-life scores within 2–4 weeks of starting abrocitinib 100 mg daily, without reported adverse events [74], while an independent case series of erythematotelangiectatic rosacea treated with the same dose documented one patient with marked clinical and vascular improvement on a tapered regimen, alongside others with only partial or absent response, including one who discontinued therapy due to a suspected reactivation of chronic hepatitis B and elevated transaminases [75]. Additional single-patient reports describe successful use of abrocitinib 100–200 mg daily in granulomatous rosacea and in an acute, intense pulsed-light-aggravated rosacea flare: in both scenarios, patients with prominent erythema, edema, and burning pain refractory to antibiotics, topical agents, and systemic corticosteroids experienced progressive resolution of inflammatory lesions over weeks to months, with maintenance on reduced or discontinued dosing and no relapse during follow-up, aside from residual scarring in the phototherapy-induced case [76,77].

Across the rosacea-spectrum disorders described the therapeutic rationale for abrocitinib converges on the central involvement of JAK1-dependent cytokine signaling in sustaining inflammation. Increased production of IL-6, IL-8, TNF-α, and IFN-γ, all of which amplify dermal inflammation via JAK/STAT signaling, is crucial in the pathogenesis. Granulomatous rosacea similarly relies on JAK-mediated cytokine loops that promote macrophage activation and granuloma formation. By selectively inhibiting JAK1, abrocitinib attenuates these upstream inflammatory circuits, reducing cytokine-driven vascular reactivity, neutrophil recruitment, and dermal immune activation, providing a unifying mechanistic explanation for clinical improvement across these diverse rosacea phenotypes [73,74,75,76,77].

#### 4.2.9. Granulomatous Dermatoses

Emerging evidence suggests that abrocitinib may have a useful role across a spectrum of cutaneous granulomatous diseases that are often refractory to standard therapies. In foreign body granulomas related to dermal fillers, including delayed-onset nodules after collagen/hyaluronic acid injections, patients with persistent facial papules and nodules unresponsive to corticosteroids and other conservative measures experienced progressive resolution of lesions over a few weeks on oral abrocitinib 100 mg daily, either as monotherapy or combined with a tapering course of systemic prednisone, without reported adverse events or relapse during short-term follow-up [78,79]. A similar JAK1-targeted strategy was applied in tattoo-related cutaneous sarcoidosis with lymph node and pulmonary involvement, where adding abrocitinib 100 mg daily to low-dose oral prednisone in a patient who had relapsed on prior steroid-based regimens led to marked improvement of eyebrow plaques and reduction in mediastinal lymphadenopathy, again without treatment-limiting toxicity [80].

Within the granuloma annulare (GA) spectrum, both localized and generalized forms appear responsive to abrocitinib. A patient with localized GA resistant to cyclosporine, hydroxychloroquine, and intralesional steroids achieved near-complete clearance of annular plaques on abrocitinib 150 mg daily, maintaining remission over several months [81], while an elderly woman with recalcitrant generalized GA responded rapidly to abrocitinib 200 mg daily with near-complete clearing of disseminated lesions and sustained control after dose tapering and discontinuation [82]. These observations are complemented by a report of long-standing pseudorheumatoid nodules—considered a subcutaneous GA variant, where abrocitinib 200 mg daily produced marked softening and eventual near-complete resolution of function-limiting nodules over six months, with recovery of joint mobility and no major safety issues [83].

The cases reported across these six articles suggest a shared inflammatory framework that may explain the observed responses to abrocitinib. Granulomatous disorders such as sarcoidosis, granuloma annulare, and foreign-body filler reactions all involve cytokine pathways, particularly interferon-γ, IL-2, and other JAK1-dependent signals, that drive macrophage activation and granuloma maintenance. The consistent clinical improvements seen with selective JAK1 inhibition therefore align with the idea that blocking these upstream cytokine circuits can dampen the cellular activation loops that sustain granulomatous inflammation. This provides a plausible rationale for the favorable outcomes observed across sarcoidosis, GA variants, deep nodular GA, and foreign-body granulomas treated with abrocitinib [78,79,80,81,82,83].

#### 4.2.10. Vitiligo

Early emerging evidence suggests that abrocitinib may offer therapeutic benefit in vitiligo. A recent case report described a 61-year-old man with active acrofacial vitiligo who had plateaued on tacrolimus, ginkgo biloba, and oral mini-pulse prednisone; switching to abrocitinib 100 mg daily resulted in rapid improvement within one month and substantial repigmentation by two months, with maintenance of response even after transitioning back to topical therapy [84].

Additional support comes from a patient with long-standing generalized vitiligo coexisting with severe atopic dermatitis, whose vitiligo had remained static despite conventional treatments. After beginning abrocitinib 100 mg daily, the patient showed progressive improvement of dermatitis alongside clear repigmentation of facial and body lesions over a four-month period [85].

A further report highlights a different clinical context: a man with stable vitiligo experienced worsening depigmentation while treated with upadacitinib for atopic dermatitis, despite complete control of eczema; switching to abrocitinib led to marked repigmentation within three months, suggesting that JAK1-selective inhibition may differentially modulate melanocyte survival pathways compared with agents with stronger JAK2 activity [86].

These reports point to abrocitinib’s targeted inhibition of JAK1-dependent signaling, particularly IFN-γ-driven pathways implicated in CD8^+^ T-cell-mediated melanocyte destruction, as a plausible explanation for its benefit [84,85,86]. Its minimal JAK2 inhibition, highlighted especially in the comparative case, may preserve melanocyte survival pathways, thereby facilitating repigmentation while dampening autoimmune inflammation [86].

#### 4.2.11. Prurigo Nodularis

Emerging reports suggest that abrocitinib may offer meaningful benefit in prurigo nodularis (PN), particularly in patients with long-standing disease refractory to conventional therapies. In two adults with decades- and years-long histories of intensely pruritic, excoriated nodules confined to the lower limbs, both unresponsive to systemic corticosteroids, immunosuppressants, antibiotics, thalidomide, and phototherapy, abrocitinib 100 mg daily led to rapid improvement within the first week and marked flattening of nodules by eight weeks, accompanied by normalization of sleep and substantial reductions in pruritus severity [87].

A third patient, with widespread non-atopic PN involving the face, hands, and posterior neck and repeatedly intolerant or unresponsive to systemic corticosteroids and thalidomide, experienced dramatic relief of pruritus within three days of starting abrocitinib 100 mg daily, with near-complete lesion regression over two months and excellent tolerability [88].

Across these cases, abrocitinib consistently provided both rapid antipruritic effects and structural improvement of nodules, even when prior systemic agents had failed or produced limiting adverse effects. Both articles highlight the central role of JAK1-dependent cytokines, particularly IL-4, IL-13, IL-22, and IL-31, in driving the neuroimmune itch–scratch cycle and keratinocyte hyperproliferation characteristic of PN. By selectively inhibiting JAK1, abrocitinib interrupts these upstream signaling pathways, offering a biologically plausible explanation for the rapid itch relief and sustained lesion improvement observed across refractory PN cases [87,88].

#### 4.2.12. Chronic Spontaneous Urticaria

Growing clinical evidence suggests that abrocitinib may represent a promising option for chronic spontaneous urticaria (CSU) that remains uncontrolled despite maximal antihistamine therapy and omalizumab escalation. In this case series, six adults with long-standing CSU, and, in several instances, comorbid allergic conditions such as rhinitis or alopecia areata, had persistent wheals, angioedema, and high Urticaria Activity Score over 7 days (UAS7) scores despite prolonged treatment with high-dose antihistamines, omalizumab at increased frequency, and various immunosuppressants including corticosteroids, methotrexate, and cyclosporine. Initiation of abrocitinib 100 mg daily resulted in rapid improvement in most patients, often within the first two weeks, with steady declines in UAS7 scores and complete remission in the majority after several months of treatment [89].

The authors highlight that cytokines involved in CSU pathogenesis, particularly IL-9, IL-10, and other mediators upstream of mast cell activation, signal through the JAK–STAT axis, with JAK1 playing a central role. Inhibition of this pathway by abrocitinib may therefore dampen aberrant mast cell activation and downstream pruritic and vascular responses, providing a biologically plausible explanation for the consistent improvements seen in antihistamine- and omalizumab-refractory CSU [89].

#### 4.2.13. Genodermatoses

Abrocitinib has recently emerged as a promising off-label therapeutic option across some genodermatoses traditionally characterized by impaired epidermal integrity, chronic inflammation, and resistance to standard treatments [90,91,92,93].

In Hailey–Hailey disease (HHD), a chronic hereditary acantholytic disorder caused by ATPase secretory pathway Ca^2+^ transporting 1 (ATP2C1) mutations, a woman with long-standing, treatment-refractory disease affecting intertriginous and perianal folds experienced rapid clinical improvement within days of initiating abrocitinib 100 mg daily, achieving complete lesion clearance within two weeks and sustained remission upon re-treatment after a brief interruption. Symptoms had previously persisted despite extensive trials of corticosteroids, methotrexate, dapsone, acitretin, and naltrexone, reflecting the severity of her condition and the notable efficacy of JAK1 inhibition in this context. In HHD, inflammation exacerbates keratinocyte fragility: modulation of IL-4/IL-13 and downstream JAK1-dependent signaling may help restore epithelial stability and reduce cytokine-driven barrier dysfunction, explaining the rapid improvement observed [90].

A similar benefit was reported in Netherton syndrome (NS), a congenital ichthyosiform disorder caused by serine protease inhibitor, Kazal type 5 (SPINK5) mutations and characterized by lymphoepithelial Kazal-type-related inhibitor (LEKTI) deficiency, barrier collapse, allergic diathesis, and extreme elevations in IgE and eosinophils. A 29-year-old woman with generalized erythroderma, hair shaft defects, and long-standing inflammation demonstrated progressive and substantial improvement over six months of abrocitinib therapy, including reductions in Body Surface Area (BSA) involvement, Eczema Area and Severity Index (EASI) scores, pruritus, and circulating eosinophils and IgE. Clinical responses persisted throughout follow-up, with no significant adverse effects. NS involves unchecked serine protease activity and profound type-2 inflammation: by inhibiting JAK1-mediated cytokine signaling, abrocitinib may suppress IL-4/IL-13-driven immune activation [91].

Additional evidence comes from Darier disease (DD), an autosomal dominant keratinization disorder associated with ATPase sarcoplasmic/endoplasmic reticulum Ca^2+^ transporting 2 (ATP2A2) mutations and impaired calcium homeostasis. In a patient with long-standing, widespread papules, crusts, and nail changes refractory to multiple treatments, combination therapy with abrocitinib and acitretin produced rapid symptomatic relief, with marked clearing of lesions within two weeks and continued improvement over 11 weeks of follow-up. While acitretin likely contributed through its antiproliferative and differentiating effects, the accelerated remission suggested an additive impact from JAK1 inhibition [93]. A second case described a 34-year-old man with genetically confirmed DD and concomitant severe atopic dermatitis who achieved near-complete resolution of both conditions within two months of abrocitinib 100 mg daily, with a dramatic reduction in pruritus and no adverse events during continued treatment [92]. IL-6 signaling and broader cytokine dysregulation appear to exacerbate DD: abrocitinib’s JAK1 selectivity may mitigate IL-6-mediated inflammatory loops that worsen keratinocyte adhesion defects [93].

#### 4.2.14. Lichen Amyloidosis

Emerging reports indicate that abrocitinib may provide a clinically meaningful therapeutic option in lichen amyloidosis (LA), a condition characterized by chronic, intense pruritus and hyperkeratotic papules that often persist despite conventional therapies. In a pilot study including two patients with recalcitrant LA limited to the shins and limbs, abrocitinib 100 mg daily resulted in rapid improvement of pruritus, dropping from scores of 8–9 to 0–1 within 2–3 weeks, accompanied by flattening of papules and progressive reduction in hyperpigmented plaques. The therapeutic benefit persisted even after dose tapering to alternate-day administration, and no adverse events were observed [94].

A complementary case report described a young man with severe AD-associated lichen amyloidosis who exhibited striking improvement in both eczema and amyloid papules on abrocitinib 100 mg daily, with pruritus decreasing from 10/10 to 1/10 within two weeks, EASI score dropping from 48 to 15 after one month, and significant flattening and lightening of LA papules noted on clinical examination. Treatment was well tolerated over several months, with sustained remission of itch and continued lesion improvement [95].

From a mechanistic standpoint, both studies highlight the central role of IL-31 and other JAK1-dependent pruritogenic pathways in LA. IL-31, which is overexpressed in affected skin and acts through JAK1/JAK2 to activate downstream STAT signaling, contributes to hypersensitivity of cutaneous nerve fibers and perpetuates the itch–scratch cycle that underlies amyloid deposition. By selectively inhibiting JAK1, abrocitinib disrupts these neurosensory and inflammatory loops [94,95].

#### 4.2.15. Other Conditions

Lastly, case reports indicate that abrocitinib may offer therapeutic benefit across a diverse set of other inflammatory dermatoses [96,97,98,99].

In eosinophilic pustular folliculitis (EPF), two adults with long-standing, treatment-refractory disease, including facial, palmar, and plantar pustules with marked eosinophilia, responded rapidly to abrocitinib 100 mg daily, achieving clearance of pustules within 1–4 weeks and maintaining remission without significant adverse effects. EPF involves Th2-skewed cytokines such as IL-4, IL-5, IL-13, and IL-31, all of which signal through JAK1; inhibition of this pathway likely suppresses eosinophilic inflammation and pruritus [96].

Similarly, acquired reactive perforating collagenosis (ARPC), a pruritic perforating dermatosis characterized by transepidermal elimination of collagen, showed marked improvement with abrocitinib 100 mg daily in a patient with persistent nodules and ulcerated papules unresponsive to numerous systemic and topical therapies. Within three months, both pruritus and lesion burden significantly decreased. ARPC lesions exhibit increased IL-4, IL-13, and IL-31 expression and a Th2-dominant infiltrate; JAK1 inhibition likely attenuates this cytokine axis, breaking the itch–scratch cycle that perpetuates collagen extrusion [97].

In persistent pityriasis rosea, a young woman with six months of treatment-resistant, pruritic, scaly trunk lesions experienced near-complete resolution after only 14 days of abrocitinib 100 mg daily, including rapid pruritus relief within the first 48 h. pityriasis rosea is driven by T-cell-mediated inflammation and cytokines such as IL-22 and other JAK1-dependent mediators; abrocitinib may blunt this inflammatory signaling and reduce associated pruritus [98].

Finally, abrocitinib has shown potential utility in eruptive pruritic papular porokeratosis (EPPP). A 75-year-old man with severe, sudden-onset pruritic papules atop a long history of porokeratosis achieved dramatic itch reduction and lesion improvement within one month of abrocitinib 100 mg daily, after multiple systemic therapies had failed. EPPP lesions demonstrate upregulation of IL-31, TSLP, periostin, and JAK1/2-dependent pruritic pathways; JAK1 inhibition rapidly reduces IL-31 activity, explaining the profound antipruritic response [99].

## 5. Results: Upadacitinib

Upadacitinib is an orally administered small-molecule Janus kinase (JAK) inhibitor with relative selectivity for JAK1. Upadacitinib received its first regulatory approval from both FDA and EMA in 2019 for the treatment of rheumatoid arthritis. Since then, its indications have been broadened to multiple immune-mediated conditions including psoriatic arthritis, ankylosing spondylitis, non-radiographic axial spondyloarthritis, Crohn’s disease and ulcerative colitis in adults, as well as atopic dermatitis in adults and adolescents aged 12 years and older in many regions. The drug is formulated as an extended-release, once-daily oral tablet available in 15, 30 and 45 mg and is marketed in the United States, Europe and numerous other countries. In addition, upadacitinib is under active investigation in clinical trials for several other inflammatory conditions and a growing body of case reports and small case series has increased interest in its off-label use for difficult-to-treat inflammatory dermatoses (Table 3) [100,101,102,103].

### 5.1. Lichenoid Dermatoses

Lichen planus (LP) is a chronic, cell-mediated inflammatory disorder affecting skin, mucosae and, in rare instances, adnexal structures [101]. Although its etiopathogenesis is multifactorial, converging evidence indicates that interferon-γ-driven cytotoxic responses and IL-21-mediated T-cell activation are core components sustaining the lichenoid inflammatory cascade, with the JAK/STAT axis representing a major intracellular signaling hub. This immunologic architecture has provided a mechanistic rationale for exploring selective JAK1 inhibition in refractory LP and its clinical variants [104]. A growing body of case-based literature has now described favorable outcomes with upadacitinib across an unexpectedly wide spectrum of lichenoid dermatoses. Beyond classical cutaneous and mucosal LP, several unusual or notoriously treatment-resistant subtypes have shown clinical responsiveness. In a patient with hypertrophic LP of long duration, upadacitinib 15 mg/day induced rapid improvement in pruritus and near-complete flattening of plaques, with remission maintained for one year [105]. A further case of unilateral blaschkoid LP also demonstrated prompt symptomatic improvement and complete clinical resolution by six months of therapy [106]. Oral LP appears similarly responsive: in a retrospective series of ten women with severe erosive disease refractory to multiple systemic agents, daily upadacitinib (15–30 mg) achieved complete or substantial mucosal clearance in most cases, with no discontinuations due to adverse events [107].

More recently, upadacitinib has shown efficacy in additional phenotypes not traditionally classified within the LP spectrum but sharing prominent lichenoid features. Keratosis lichenoides chronica (KLC), a notoriously recalcitrant dermatosis characterized by linear and reticulated keratotic papules, responded remarkably in a 30-year-old man who obtained near-complete clearing after five months of therapy, following failure and intolerance of acitretin [108]. Similarly, isolated nail involvement, often difficult to treat due to matrix fibrosis and limited drug penetration, was successfully managed with upadacitinib, with progressive normalization of the nail plate documented in the available report [109]. Evidence has also expanded through a detailed case series describing the effects of upadacitinib across diverse LP variants, including LP pigmentosus, generalized cutaneous LP, hypertrophic LP, and lichen planopilaris (LPP). All patients exhibited histologically confirmed lichenoid dermatitis and had failed multiple topical and systemic therapies; nonetheless, upadacitinib, typically initiated at 30 mg and tapered to 15 mg, led to meaningful clinical improvement in each case. Responses encompassed resolution of pruritus, fading of violaceous or hyperpigmented patches, reduction in perifollicular scaling in LPP, and even early hair regrowth in scarring alopecia. Importantly, improvements were sustained despite dose de-escalation in long-term follow-up [110].

Additional interface dermatoses outside the LP spectrum have also been reported to respond. In a bullous pityriasis lichenoides et varioliformis acuta (PLEVA) case with a polymorphic course and histologic confirmation after multiple prior biopsies, upadacitinib 15 mg/day led to rapid clearance within one month after failure of topical corticosteroids and topical ruxolitinib and worsening on deucravacitinib and dupilumab; no treatment-limiting adverse events were attributed to upadacitinib, although therapy was later stopped during hospitalization for a breakthrough seizure related to underlying epilepsy [111].

### 5.2. Psoriasis

In a case series of five male patients with psoriasiform eczema complicated by distinct immune-mediated comorbidities, including alopecia areata, vitiligo, ulcerative colitis, and hidradenitis suppurative, treatment with upadacitinib (15–45 mg/day) led to rapid and consistent improvement of cutaneous inflammation. Responses in comorbid conditions varied but were generally favorable: alopecia areata showed early regrowth, vitiligo exhibited partial repigmentation, and ulcerative colitis achieved clinical and endoscopic remission, all without reported adverse events [112].

Additional evidence derives from two cases of severe palmoplantar plaque psoriasis, a notoriously resistant phenotype often refractory to retinoids, phototherapy, and multiple biologic classes. Both patients achieved complete clearance of palmar and plantar plaques within three months on upadacitinib 15 mg/day, suggesting a previously underappreciated role for JAK1 signaling in the pathophysiology of palmoplantar psoriasis [113]. Upadacitinib has also shown efficacy in situations involving paradoxical psoriatic eruptions. A patient with longstanding atopic dermatitis who developed dupilumab-induced psoriasis showed complete clearance following transition to upadacitinib 15 mg/day, with sustained disease control for over a year and no adverse events [114]. Another case highlights its utility in nail-limited psoriasis, an entity frequently resistant to standard therapies, where a 13-year-old patient with severe nail involvement achieved near-complete remission within 20 weeks on upadacitinib monotherapy [115].

In two reported cases of co-existent psoriasis and allergic contact dermatitis (ACD), upadacitinib produced rapid and durable control after complex treatment courses. In the first case (50-year-old man), psoriasis improved on IL-17 blockade, but an eczematous eruption emerged; dupilumab improved eczema but psoriasis later recurred. Patch testing identified multiple fragrance-related allergens (including Balsam of Peru and fragrance mix), with ongoing household exposure limiting avoidance. After stopping dupilumab, upadacitinib 15 mg daily achieved complete clearance within 1 week and maintained remission of both phenotypes through 24 months. In the second case (45-year-old man) with biopsy-proven psoriasis, methotrexate and then certolizumab were followed by development of vesicular hand dermatitis and widespread eczema (spongiotic dermatitis with eosinophils); dupilumab cleared truncal eczema but hand/forearm disease persisted, and patch testing was positive for fragrance mix and lavender with relevant exposure. Switching to upadacitinib 15 mg daily led to complete clearance by 2 months with sustained response at 12 months; one episode of herpes zoster occurred and resolved with valacyclovir [116]. Lastly, a multicenter case series further reported that seven patients with biopsy-proven psoriasiform and spongiotic dermatitis treated with upadacitinib (15–30 mg/day) for at least 16 weeks experienced consistent improvement in global severity, body surface area involvement, and pruritus, supporting a role for JAK inhibition in this diagnostically challenging overlap phenotype [117].

### 5.3. Neutrophilic and Eosinophilic Dermatoses

Pyoderma gangrenosum (PG) is an uncommon neutrophilic disorder that typically manifests with rapidly progressive, painful ulceration. Lesions often begin as inflammatory papules or pustules and evolve into ulcers with undermined violaceous borders. Clinical variants (such as bullous, vegetative, peristomal) are recognized. Although multiple local and systemic therapies have been utilized for PG, high-quality efficacy studies are lacking for most interventions. Topical corticosteroids, systemic corticosteroids, and cyclosporine are common initial therapies [118]. In a 65-year-old woman with HLA-B27-negative spondyloarthritis and therapy-resistant PG, upadacitinib 15 mg/day led to a marked clinical improvement by 6 weeks, complete remission by 12 weeks, and sustained inactivity through 24 weeks; treatment was well tolerated and allowed prednisone tapering to 3 mg/day [119]. In a separate facial PG case (20-year-old man) triggered after excision of facial cysts and unresponsive to antibiotics and debridement, initial oral prednisolone 0.5 mg/kg once daily for 2 weeks was followed by escalation to upadacitinib 15 mg once daily with a tapering prednisolone course; the ulcers showed substantial healing within about 1 month and complete resolution by 4 months, with no adverse events reported [120].

In case of chronic, steroid-dependent Sweet syndrome, a 75-year-old man had recurrent neutrophilic dermatosis controlled only with prednisone 20 mg/day, with repeated flares on tapering and significant long-term corticosteroid toxicity. After an unsuccessful trial of mycophenolate mofetil, upadacitinib was introduced initially 15 mg/day with prednisone 20 mg/day, then escalated to 30 mg/day at 1 month and to 45 mg/day by month 7, enabling progressive prednisone tapering. By 12 months the eruption had completely cleared, and prednisone was stopped. When upadacitinib was later reduced to 30 mg/day after sustained remission, lesions recurred within a month and control was re-established after resuming 45 mg/day [121].

A 52-year-old man was diagnosed with papuloerythroderma of Ofuji after several months of pruritic red papules on the trunk that progressed to an erythrodermic eruption with characteristic fold-sparing (“deck-chair sign”). Histology was consistent with a spongiotic dermatitis pattern with eosinophils and laboratory exams showed peripheral eosinophilia with normal total IgE. After limited improvement with topical steroids he started upadacitinib 30 mg daily and marked clinical improvement after 4 weeks was reported. After 6 months, the dose was tapered to 15 mg once daily, and the eruption remained well controlled with no recurrence during an additional 8 months of monitoring with no adverse events reported [122].

A further case of a 16-year-old male diagnosed with generalized eosinophilic pustular folliculitis had a 2-year, progressively generalized, intensely pruritic folliculo-centric eruption refractory to topical and systemic corticosteroids and only mildly responsive to methotrexate. Work-up showed peripheral eosinophilia and elevated total IgE, while biopsy demonstrated follicular spongiosis with prominent eosinophilic infiltration. Tofacitinib produced rapid symptomatic and clinical improvement with normalization of eosinophils and IgE (and reduction in measured Th2-related cytokines), but disease relapsed on dose reduction and then became unresponsive despite re-escalation. He was then switched to upadacitinib which led to complete clearance within 4 weeks and maintained remission through 6 months of follow-up with no reported adverse events [123].

### 5.4. Follicular Occlusion Disorders

Across follicular occlusion disorders, emerging case-based evidence suggests that upadacitinib may provide clinically meaningful benefit in refractory hidradenitis suppurativa (HS) and dissecting cellulitis of the scalp (DCS), including patients previously failing biologics. HS is a chronic, disabling inflammatory skin disease characterized by recurrent painful nodules, abscesses, and sinus tracts. The precise etiology of HS remains incompletely understood, but it is thought to involve a combination of genetic, immunological, and environmental factors [124]. DCS is a rare condition characterized by painful inflammatory nodules and abscesses on the scalp, often leading to sinus tracts and scarring alopecia [125]. In a severely obese man with treatment-refractory HS and elevated inflammatory markers, escalation to upadacitinib 45 mg daily was associated with rapid symptomatic improvement (reduced pain, drainage, and flares) and objective disease improvement as the International Hidradenitis Suppurativa Severity Score System (IHS4) decreased from 11 to 4, with parallel reductions in systemic inflammation over 2 months, without reported adverse events [126]. In another recalcitrant HS case, upadacitinib 30 mg daily was added to ongoing adalimumab 80 mg weekly, yielding improvement at 2 months (IHS4 13 → 8); after stopping adalimumab and increasing to upadacitinib 45 mg daily, further improvement was documented by 4 months (IHS4 4) without reported adverse events [127]. A separate report described a woman with longstanding, severe HS refractory to adalimumab and later secukinumab (with paradoxical psoriasis on adalimumab and worsening HS on secukinumab), in whom upadacitinib 15 mg daily led to rapid systemic symptom relief and marked HS improvement by 28 weeks (IHS4 36 → 4), again without reported adverse events [128]. Finally, in DCS refractory to conventional therapy, upadacitinib (reported as 15 mg twice daily) was associated with substantial reductions in pain, drainage, and bleeding within 1 month and further clinical improvement by 2 months, with no major side effects reported in short follow-up [125].

### 5.5. Pityriasis Rubra Pilaris

Pityriasis rubra pilaris (PRP) is a rare papulosquamous inflammatory dermatosis characterized by follicular hyperkeratotic papules. Although its pathogenesis remains incompletely defined, increasing evidence implicates IL-23/Th17-skewed inflammation, which has prompted off-label use of biologics; however, refractory disease remains common, and the rationale for JAK inhibition is still evolving [101].

An 81-year-old woman with near-erythrodermic PRP and comorbid cardiovascular disease, previously treated with systemic corticosteroids (with rebound), failed dupilumab and subsequently had no appreciable improvement on acitretin plus ixekizumab. The switching to upadacitinib 15 mg/day led to near-complete clearance by week 4 (<1% BSA) with complete resolution of pruritus and notable hair regrowth, without laboratory deterioration (liver enzymes remained normal) and with ongoing disease control on therapy [129].

A 26-year-old woman with long-standing PRP refractory to topical agents, isotretinoin and phototherapy achieved only partial benefit on ustekinumab and worsened on ixekizumab; initiation of upadacitinib 15 mg/day produced 25% improvement by day 14, prompting escalation to 30 mg/day, after which a 65% overall improvement was documented by week 6; treatment was generally well tolerated with headache as the only reported adverse effect, and the patient later elected to transition to guselkumab for preference toward intermittent injections [130].

Finally, in a two-patient report of erythrodermic PRP with inadequate response to long-term secukinumab, both patients were switched to upadacitinib 15 mg/day: a 42-year-old man experienced complete clearance of neck and trunk involvement by 24 weeks with residual palmoplantar keratoderma, while a 13-year-old girl demonstrated marked improvement by 4 weeks and was almost completely cleared by week 24; tolerability was favorable, with acne noted in the pediatric patient [131].

### 5.6. Connective Tissue Diseases

Cutaneous lupus erythematosus (LE) is commonly framed as an interferon-driven autoimmune dermatosis. Tissue damage can lead to the release of endogenous nucleic acids (e.g., DNA/RNA fragments), which act as danger signals and promote activation of epidermal and immune cells, particularly plasmacytoid dendritic cells, resulting in increased type I interferon production. Type I interferons then engage their receptors on keratinocytes and propagate signaling via the JAK/STAT axis, inducing a pro-inflammatory transcriptional program that includes chemokines such as CXCL10. CXCL10 supports recruitment of C-X-C motif chemokine receptor 3 (CXCR3)-expressing immune cells into the skin, amplifying local inflammation and contributing to ongoing keratinocyte injury, including apoptotic and necrotic cell death. Consistent with this pathogenic model, selective JAK1 inhibition has been proposed to attenuate interferon-associated signaling and downstream immune activation [132]. In a case report, a 26-year-old woman with discoid lupus erythematosus was treated with upadacitinib 15 mg/day as monotherapy. She experienced rapid and sustained improvement over 28 weeks. Dose reduction to alternate-day dosing led to relapse, which improved again after resuming daily therapy. Acne was reported as the main adverse event (managed topically) and no significant laboratory toxicity was observed during follow-up [133].

As in LE, evidence from case reports suggests that upadacitinib can be effective in other connective tissue diseases, including refractory amyopathic dermatomyositis (ADM) and diffuse cutaneous systemic sclerosis (dcSSc). ADM is an uncommon variant within the dermatomyositis spectrum in which patients exhibit the characteristic cutaneous features of dermatomyositis but lack clinically significant muscle involvement for a defined interval. Experimental data from both laboratory models and patient-based studies support a central role for type I interferon-mediated signaling in DM. In line with this, JAK1/2 inhibitors have been shown to interfere with type I interferon-driven injury in muscle and endothelial cells [134]. In anti-melanoma differentiation-associated gene 5 antibody (anti-MDA5)-positive ADM without interstitial lung disease, a 35-year-old woman with refractory cutaneous disease despite multiple immunomodulators, achieved complete clinical remission after 6 weeks on upadacitinib 30 mg/day, with early pruritus improvement and a reduction in anti-MDA5 antibody titer, without reported adverse events over 6 months of follow-up [135]. A second refractory ADM case (69-year-old man) presented with severe pruritus and typical cutaneous signs (heliotrope erythema, Gottron lesions, and photo-distributed erythema such as the V-sign/shawl sign). The disease was highly refractory despite multiple prior systemic therapies (including corticosteroids, methotrexate, hydroxychloroquine, rituximab, IVIG, apremilast, and mycophenolate), with persistent active skin disease and marked itch impairing quality of life. Upadacitinib was initiated at 15 mg/day with only partial response; after escalation to 30 mg/day as monotherapy, the itch resolved within a week, and complete cutaneous remission was achieved within about one month, remaining stable at 6 months without reported adverse events [136]. Similarly, a 66-year-old woman with ADM who had an incomplete response to baricitinib showed substantial improvement in skin clearance and itch reduction after 3 months of upadacitinib 30 mg/day and remained almost clear at 6 months, without notable laboratory changes [137].

Systemic sclerosis (SSc) is a complex autoimmune disease driven by vasculopathy, immune dysregulation, and fibrosis, leading to fibroblast activation and excess collagen deposition with frequent multisystem involvement. In a long-standing, multisystem dcSSC (Scl-70 positive), a 52-year-old woman with a 14-year disease history with prominent vascular and cutaneous burden (Raynaud phenomenon with digital ulceration and auto-amputation, sclerodactyly, and extensive calcinosis requiring multiple surgeries), as well as internal organ involvement (esophageal scleroderma and interstitial lung disease), had failed multiple therapies including nintedanib, sevelamer, colchicine, tadalafil, and prior prednisone courses. She also discontinued mycophenolate mofetil following a postoperative infection. She therefore received upadacitinib 15 mg for 2 months with immediate symptomatic and clinical improvement. She then experienced a significant flare during a 1-month interruption, and improvement recurred after restarting. A transient AST elevation occurred and self-resolved, probably attributed to a viral infection [138].

### 5.7. Photodermatoses

Evidence for upadacitinib in photodermatoses remains limited but is growing through individual reports and small series, suggesting rapid relief of inflammation-driven burning and pruritus in severe, treatment-refractory disease. In chronic actinic dermatitis, a 75-year-old man with histologically confirmed disease was refractory to topical and systemic steroids, calcineurin inhibitors and ciclosporin. He achieved early itch improvement within 2 weeks on upadacitinib 15 mg daily and complete clinical resolution by week 4, with sustained remission over 8 months and no adverse events reported [139].

A larger signal comes from a three-patient series of severe chronic photodermatitis in which all three male patients treated with upadacitinib 15 mg twice daily experienced marked improvement within 2 weeks, including dramatic reductions in burning and itch scores and durable control during follow-up. Tolerability was generally favorable, although one patient gained 4 kg within the first 2 months [140].

Finally, in pediatric actinic prurigo, a 12-year-old boy with HLA-DRB1*0407 positivity and severe summer flares despite multiple systemic and biologic therapies showed rapid improvement on upadacitinib 15 mg daily without reported side effects. Notably, missing a dose during sun-exposure triggered relapse, with symptom relief returning within hours after resuming treatment [141].

### 5.8. Vasculopathies

Almaghrabi et al. describe a 39-year-old woman with biopsy-confirmed urticarial vasculitis. Her lesions were painful and lasted > 24 h. She also had systemic symptoms, including arthralgia, myalgia, fatigue, and blurred vision. Multiple prior therapies had failed, including steroids, colchicine, omalizumab, dapsone, rituximab, mycophenolate, and cyclosporine. Upadacitinib was started at 30 mg once daily. Concomitant dapsone and a dose of omalizumab were also given, and prednisolone was tapered. Within 1 month, skin lesions were nearly completely cleared. Systemic symptoms also improved markedly. No adverse events were reported, and no serious safety signals were noted during follow-up [142]. Another case involves a 54-year-old woman with livedoid vasculopathy and painful lower limbs ulcerations. Histology supported the diagnosis, showing thrombotic changes in superficial dermal vessels. Prior treatments were ineffective. Upadacitinib 15 mg/day was initiated, and pain improved dramatically by day 1. Visible skin improvement began within the first week. Near-complete ulcer healing was documented by day 52. The only reported adverse event was mild herpes labialis which resolved without complications. No laboratory abnormalities were detected [143].

### 5.9. Bullous Pemphigoid

Bullous pemphigoid (BP) is an autoimmune subepidermal blistering disease driven by pathogenic immune responses against components of the dermal–epidermal junction. Immunologic studies increasingly implicate T-helper polarization abnormalities, with a Th1/Th2 disequilibrium and a strong Th2-associated signature in BP lesions and peripheral blood, including elevated activity of IL-4 and IL-13, which promote eosinophilic inflammation and IgG/IgE-mediated pathways relevant to blister formation [144].

Su et al. report a 66-year-old man with moderate bullous pemphigoid and an over 10 years history of psoriasis that had been clinically quiescent. He initially improved on systemic methylprednisolone, but after discharge he was reluctant to continue glucocorticoids and was transitioned to dupilumab with gradual steroid tapering. While bullous pemphigoid came under control, psoriasis developed and progressed during dupilumab therapy. Upadacitinib was then started at 15 mg once daily. After 2 weeks, bullous pemphigoid was effectively controlled, and the psoriatic eruption began to regress. By week 4 both conditions had improved substantially, and after week 10 the patient achieved complete clearance without recurrence (BP involvement was <5%). No upadacitinib-related adverse events were reported in the case [145]. Another case describes a 74-year-old woman who developed drug-induced bullous pemphigoid during immunotherapy (MK-4830 plus pembrolizumab) for metastatic programmed death-ligand 1 (PD-L1)-positive head and neck squamous cell carcinoma. The eruption started as intensely pruritic urticarial plaques. It then progressed to widespread tense bullae shortly after a subsequent treatment cycle. This occurred despite prolonged high-dose systemic corticosteroids and topical steroids. Because the disease remained refractory and the patient transitioned to palliative care, upadacitinib was started off label at 15 mg/day. At 4 weeks, the urticarial component was improving and erosions were healing. No active bullae were seen, and there was no mucosal progression. No adverse events attributed to upadacitinib were reported. Unfortunately the patient passed away from her malignant tumor about one month later [144].

### 5.10. Chronic Prurigo

Chronic prurigo is a neuroinflammatory skin disease defined by pruritus occurring for over 6 weeks. Compared to non-lesional areas, lesional skin displays an increased expression of Type 2 cytokines, such as IL-4, IL-13, IL-31. This pattern explains the benefit observed with JAK inhibition [101].

A 53-year-old man with refractory prurigo nodularis had persistent pruritic hyperkeratotic nodules with sleep impairment and high inflammatory markers (mild eosinophilia and elevated IgE). After partial response or intolerance to multiple systemic therapies (including cyclosporine, prednisone, methotrexate, and off-label dupilumab), upadacitinib was started off-label at 15 mg twice daily. Clinical improvement was evident within the first month, and by week 16 the patient achieved complete clearance without laboratory abnormalities or reported adverse events [146]. In a real-life case series of 3 patients with chronic prurigo (papular/nodular and plaque subtypes) who had severe itch and major quality-of-life impairment despite prior treatments (topical/intralesional corticosteroids, gabapentinoids, methotrexate, and/or cyclosporine), upadacitinib 15 mg once daily produced very rapid antipruritic effects. All patients reported itch improvement within 3–7 days, with complete itch control by week 2, followed by gradual clearance of lesions. No relapses or adverse events were observed during a median follow-up of about 6 months [147].

### 5.11. Granuloma Annulare

Granuloma annulare (GA) is a non-infectious inflammatory skin disease characterized by granulomatous inflammation. Recent studies suggest that GA displays an increased Th1 and Th2-associated immune activity with evidence of JAK-STAT pathway activation. IL4 appears to be particularly increased [148].

A 57-year-old woman developed biopsy-confirmed generalized GA. Multiple prior treatments had failed, including NB-UVB, high-potency topical steroids, monthly rifampicin–ofloxacin–minocycline (ROM) therapy, intralesional triamcinolone, and topical ruxolitinib. Upadacitinib was started at 15 mg once daily. Near-complete clearance was documented within 4 weeks. She remained clear on maintenance therapy at the time of reporting. No adverse events were reported [149]. Another case describes 2 women with long-standing, refractory generalized GA, who were treated with upadacitinib 15 mg once daily after extensive prior therapy failures. Previous regimens included combinations of ROM therapy, potent topical steroids, dapsone, hydroxychloroquine, NB-UVB, pentoxifylline, methotrexate, and apremilast (varying by case). In the first case (11-year history), improvement became evident by 8 weeks. Complete clearance was achieved by 4 months. Remission persisted at 6 months while continuing 15 mg daily. In the second case (21-year history), partial improvement was noted by 3 weeks. Complete clearance was reached by 2 months. A mild headache occurred in this patient. No other adverse events were reported [150]. Lastly, a 53-year-old man with generalized papular granuloma annulare had persistent papular disease without typical plaques. He had not responded to topical and systemic corticosteroids, methotrexate, or NB-UVB. Upadacitinib was started at 15 mg once daily. Lesions began to thin within 2–4 weeks. The dose was then tapered (every other day, then every 2 days) and stopped. Complete remission was achieved by 8 weeks overall. No relapse was reported 6 months after discontinuation. No adverse effects were reported [151].

### 5.12. Vitiligo and Alopecia Areata

Vitiligo is a common, chronic depigmenting disorder in which immune dysregulation results in the acquired destruction of melanocytes. Lesional skin often shows an enrichment of melanocyte-specific cytotoxic CD8^+^ T cells, which can mediate melanocyte loss via effector mechanisms. Epidermal keratinocytes also participate in sustaining inflammation by producing IFN-γ, thereby inducing CXCR3 ligands such as C-X-C motif chemokine ligand 9 (CXCL9) and CXCL10. These chemokines facilitate continued trafficking of CD8^+^ T cells to the skin, reinforcing local immune activation and promoting the spread of melanocyte injury beyond the original focus. Accordingly, selective JAK1 inhibition could theoretically attenuate IFN-γ-dependent signaling and help limit disease progression [152].

Alopecia areata (AA) is a common inflammatory, non-scarring condition involving an autoimmune process targeting hair follicles, influenced by genetic susceptibility and environmental triggers. Standard therapies (topical and intralesional steroids, minoxidil, and systemic immunosuppression) have variable effectiveness and can cause adverse effects or relapses. Increasing evidence links AA to cytokine-driven immune activation involving interferons, interleukins, and cytotoxic CD8^+^ T cells, which signal through the JAK/STAT pathway, providing a rationale for JAK inhibition as a newer therapeutic strategy [153].

A 9-year-old girl with refractory segmental vitiligo and concomitant alopecia areata showed poor response to prednisone plus topical therapy. She was then treated with upadacitinib 15 mg once daily combined with NB-UVB and a topical calcineurin inhibitor. Repigmentation started within 1 month and continued to improve over time, with about 70% improvement by month 7 and visible hair regrowth in alopecia lesions. A transient, asymptomatic creatine kinase rise was observed and normalized spontaneously with monitoring [154]. In a single-center retrospective series of 10 patients with nonsegmental vitiligo, most were treated with upadacitinib 30 mg daily (one 15-year-old received 15 mg). Repigmentation was observed in essentially all patients with available follow-up, with improvement already measurable at week 12 and sustained in those followed up with to week 24. Safety was generally favorable; one patient developed herpes simplex virus type 1 (HSV-1) gingivostomatitis without needing drug discontinuation, and another had an elbow carbuncle after an insect bite that required brief drug interruption and antibiotics [155]. A 67-year-old man with abrupt alopecia universalis that was refractory to intralesional and oral steroids was treated with upadacitinib 15 mg. He began responding by around 2–3 months, and achieved complete regrowth by week 16, with improvement in nail dystrophy as well. He later stopped therapy administration and maintained regrowth for about 6 months, then relapsed after a major stressor and regained full regrowth after restarting upadacitinib. The only reported laboratory issue was a transient, self-resolving elevation of amylase and lipase [156]. Five patients with alopecia areata of different severities, mostly with Severity of Alopecia Tool (SALT) 100, received upadacitinib 15 mg once daily after failing conventional treatments. Most showed progressive SALT improvement by 1–3 months, and 4 out of 5 patients reached SALT 0 by week 24. No adverse events were reported in this case series, and the authors describe routine baseline screening and follow-up safety monitoring [153].

### 5.13. Other Conditions

Hailey–Hailey disease was reported in a 65-year-old woman with long-standing, biopsy-confirmed disease and painful intertriginous erosions. Multiple prior therapies had been ineffective or poorly tolerated (including several topical/systemic agents). Upadacitinib 15 mg once daily led to healing within about 4 weeks. She remained clear through 16 weeks, with normal follow-up labs and no notable adverse events reported [157]. Cutaneous sarcoidosis (lupus pernio phenotype) was described in a 56-year-old man with very refractory facial disease despite multiple systemic treatments over the years. Upadacitinib 15 mg daily was associated with near-complete cutaneous clearance by 6 months and improvement in inflammatory symptoms. Disease worsened after stopping and improved again after restarting, supporting a drug–response relationship [158]. An 87-year-old man with diffuse erythroderma and severe pruritus was initially treated as severe dermatitis and started on upadacitinib 15 mg daily. Two months later, mycosis fungoides stage 3 (T4N0M0B0) was confirmed on repeat biopsies. Despite the malignant diagnosis, he showed marked clinical improvement on upadacitinib, with major reductions in erythema, scale, pruritus, and involved body surface area that persisted at 16 weeks. Elevated triglycerides were noted, while other labs were described as normal [159]. A 67-year-old man with Trichophyton rubrum tinea corporis developed a marked id reaction after the antifungal therapy improved the primary infection. His symmetric eczematous eruption did not respond to topical steroids or antihistamines, and he refused to use systemic steroids. Upadacitinib 15 mg/day led to progressive improvement and major control by around 8 weeks [160]. Cutaneous pseudolymphoma with an IgG4-rich infiltrate was reported in a 41-year-old woman with slowly progressive disfiguring facial nodules. Prior therapies (including steroids and antimalarials) gave only temporary or incomplete benefit. Upadacitinib 30 mg/day led to visible softening and reduction in nodules within 4 weeks and continued improvement over months, without adverse effects reported [161]. Stevens–Johnson syndrome/toxic epidermal necrolysis (SJS/TEN) overlap was treated in a 54-year-old woman using methylprednisolone plus upadacitinib 15 mg/day for 2 weeks. Progression halted quickly, and rapid re-epithelialization followed, with complete healing reported within the subsequent weeks. No treatment-related adverse events were described [162]. Persistent erythema multiforme with mucosal involvement occurred in a 56-year-old woman with follicular lymphoma and was refractory to multiple immunomodulatory approaches. Upadacitinib 15 mg daily together with low-dose prednisone achieved near-complete skin clearance within about 9 weeks and maintained mucosal control. Brief treatment interruptions were followed by prompt symptom recurrence in one instance, improving again on restart [163]. Severe chronic pruritus related to polycythemia vera was described in a 40-year-old woman. Several antipruritic strategies failed, and dupilumab provided only a transient benefit. Upadacitinib 15 mg daily led to marked itch reduction by 2 months and sustained control by 6 months; acneiform facial lesions were the main reported adverse event [164]. Refractory rosacea was reported in 2 women treated with upadacitinib. Both had erythemato-telangiectatic rosacea with prominent flushing and symptoms that persisted despite extensive conventional and adjunctive therapies. Upadacitinib was given at 15 mg once daily. One patient noticed improvement within 48 h and had clear benefit by 2 weeks; dose spacing triggered a brief rebound that stabilized with a slower taper. The second patient improved by week 4, then was lost to follow-up. No adverse events were reported [75]. A case of adult-onset Still’s disease was reported in a patient presenting with fever, arthralgia, and widespread, intensely pruritic erythematous papules and patches that were refractory to conventional therapy. Initial treatment with high-dose methylprednisolone, methotrexate, NSAIDs, and antihistamines controlled systemic symptoms but left the pruritic skin lesions unchanged. Baricitinib 1 mg twice daily was then introduced with partial improvement but was stopped because of mild creatinine elevation. Therefore, upadacitinib 15 mg once daily was introduced. The cutaneous lesions and laboratory abnormalities completely resolved within about 1 month, and glucocorticoids were successfully tapered. During 12 months of JAK inhibitor therapy, no clinically significant adverse events were observed [165].

**Table 3:** Summary of published case reports and case series describing off-label dermatologic uses of upadacitinib. Clinical indications marked with an asterisk (*) represent conditions for which the drug is currently being investigated in clinical trials. Underlined clinical indications indicate the main off-label condition for which the drug was used. **Bold** was used to highlight case series.

First Author/Year	Indication	Study Design	Number of Patients	Drug/Dose/Formulation/Association	Treatment Duration	Follow-Up	Previous Systemic Treatments	Clinical Outcome	Adverse Events/Safety
Magdaleno-Tapial et al., 2024 [155]	Nonsegmental vitiligo *	**Case series**	10 (9 adults, 1 adolescent)	Upadacitinib 30 mg QD PO (15 mg for 1 pediatric case)	12–24 weeks	24 weeks	Variable (only 1 on phototherapy)	90% showed VES improvement; facial repigmentation up to 80%	No AEs
Gil-Lianes et al., 2024 [147]	Chronic prurigo * (papular/nodular and plaque types)	**Case series**	3 (2 F, 1 M, 32–69 y)	Upadacitinib 15 mg QD PO	5–7 months	Median 6 months	Topical/intralesional corticosteroids; antihistamines/gabapentinoids; methotrexate; cyclosporine (varied by case)	Peak pruritus improved within 3–7 days with complete itch resolution by 2 weeks; lesions cleared within ~3–8 weeks with residual pigmentary change	No AEs
He et al., 2024 [153]	Alopecia areata *	**Case series**	NR	Upadacitinib (dose NR) PO	NR	NR	NR	Clinical improvement reported across cases (per title)	No AES
Noot et al., 2025 [107]	Oral lichen planus	**Case series**	10 (F, mean age 68)	Upadacitinib 15–30 mg/day	NR	NR	Multiple systemic failures (steroids, cyclosporine, methotrexate, azathioprine)	Marked improvement/near clearance	No AEs
Shahriari et al., 2024 [117]	Psoriasiform and spongiotic dermatitis (biopsy-proven)	**Multicenter case series**	7 (6 F, 1 M; 16-69 y)	Upadacitinib 15–30 mg QD PO	≥16 weeks	16 weeks	Failed topical and/or systemic therapies (incl. multiple biologics/DMARDs in several cases)	All patients improved after 16 weeks (PGA decreased to 0–2; BSA reduced; pruritus complete/near-complete resolution)	No AEs
Salvi et al., 2024 [112]	Psoriasiform eczema with immune-mediated comorbidities	**Case series**	5 (5 M, 35–64 y)	Upadacitinib 30 mg QD → 15 mg QD maintenance	≥6 months	≥1 year	Topicals, acitretin, various	Rapid improvement; UC remission; improved AA and HS	No AEs
McNamara & Tjahjono, 2025 [110]	Cutaneous lichen planus and variants	**Case series** + review	4 (3 F, 1 M, NR)	Upadacitinib 30 mg/day → 15 mg/day or intermittent dosing	Up to 9+ months	Up to 9 months	TCS, TCI, phototherapy, HCQ, MMF, MTX, antibiotics, corticosteroids	Significant improvement to clearance; hair regrowth in LPP; sustained response on lower dose	Acne; transient liver enzyme elevation
Al-Marri et al., 2025 [140]	Chronic photodermatitis (chronic photodermatosis)	**Case series**	3 (3 M, 33–56 y)	Upadacitinib 15 mg BID PO (case 2 with topical tacrolimus)	NR (reported follow-up ranged 2.5–8 months)	Up to 8 months	Topical steroids/TCIs; systemic steroids; ciclosporin; hydroxychloroquine; dupilumab (varied by case)	Marked improvement by 2 weeks in all cases with sustained symptom control and reduced burning/itch scores	Weight gain (+4 kg) in 1 case; no AEs reported in 1 case; NR in 1 case
Ch’en et al., 2024 [116]	Co-existent allergic contact dermatitis and psoriasis	**Case series**	2 (M, 50 y; M, 45 y)	Upadacitinib 15 mg QD PO (monotherapy)	12–24 months	12–24 months	Multiple biologics and MTX; dupilumab (both cases) and prior psoriasis biologics	Complete clearance (case 1 within 1 week; case 2 by 2 months) with sustained control on maintenance	Herpes zoster (case 2) resolved with valacyclovir; otherwise well tolerated
Li et al., 2024 [131]	Erythrodermic pityriasis rubra pilaris	**Case series**	2 (M, 42 y; F, 13 y)	Upadacitinib 15 mg QD PO	6 months	6 months	Acitretin; secukinumab	Rapid and near-complete clearance in both cases	No AEs
Choi et al., 2025 [113]	Palmoplantar plaque psoriasis	**Case series**	2 (F, 61 y; F, 52 y)	Upadacitinib 15 mg QD PO	3 months	Ongoing	Topicals, phototherapy, acitretin, secukinumab, apremilast, ustekinumab, etc.	Complete response within 3 months	Slight ↑ triglycerides (case 1)/↑ ALT (case 2); no clinical AE
Zhang et al., 2024 [75]	Refractory erythematotelangiectatic rosacea	**Case series** + review	2 (F, 28 y; F, 45 y)	Upadacitinib 15 mg QD PO	12 weeks	12 weeks	Multiple topicals and systemic treatments (minocycline, HCQ, etc.)	Marked improvement in erythema, flushing, pruritus within 48 h; maintained response	No AEs
Islam Z., Ch’en P. Y. et al., 2025 [126]	Refractory hidradenitis suppurativa *	Case report	1 (M, 38 y)	Upadacitinib 45 mg QD PO (with concomitant topical/antibiotic regimen as per case)	2 months	2 months	Infliximab (adverse reaction); double-dose adalimumab; multiple antibiotics and adjuncts	Marked improvement in pain/drainage/flares; IHS4 11 → 4; CRP and IL-6 decreased	No AEs
Islam Z., Choi S. et al., 2025 [127]	Refractory hidradenitis suppurativa *	Case report	1 (M, 25 y)	Upadacitinib 30 mg QD PO added to adalimumab 80 mg weekly; then upadacitinib 45 mg QD PO monotherapy	4 months	4 months	Infliximab; adalimumab (standard then intensified); antibiotics/topicals; finasteride	Improved drainage/inflammation; IHS4 13 → 8 at 2 months and 4 at 4 months	No AEs
Takei et al., 2025 [128]	Refractory hidradenitis suppurativa *	Case report	1 (F, 50 y)	Upadacitinib (dose not reported)	NR	NR	Adalimumab; secukinumab	Marked clinical improvement	No AEs
Muntaner-Virgili et al., 2024 [146]	Prurigo nodularis * (refractory)	Case report	1 (M, 53 y)	Upadacitinib 15 mg BID PO (off label)	16 weeks	16 weeks	Acitretin; intralesional triamcinolone; potent topical corticosteroids; cyclosporine (stopped for AEs); oral prednisone; methotrexate; dupilumab (off label)	Rapid improvement of itch and nodules; IGA improved to 1 at 1 month and complete clearance by week 16 (BSA 0%; WI-NRS 0)	No AEs
Mu et al., 2024 [154]	Segmental vitiligo and alopecia areata (pediatric) *	Case report	1 (NR)	Upadacitinib + NB-UVB (dose NR)	NR	NR	NR	Successful treatment (per title)	No AEs
Schundler et al., 2025 [105]	Hypertrophic lichen planus	Case report	1 (M, NR)	Upadacitinib 15 mg QD	NR	NR	Multiple systemic failures	Marked improvement	No AEs
Rosenbaum et al., 2024 [106]	Unilateral blaschkoid lichen planus	Case report	1 (F, 48 y)	Upadacitinib 15 mg QD PO	6 months	6 months	Topicals; prednisone; methotrexate; NB-UVB	Near-complete resolution; worsening on alternate-day dosing	No AEs
Tang J. et al., 2024 [108]	Keratosis lichenoides chronica	Case report	1 (M, 30 y)	Upadacitinib 15 mg QD PO	5 months	5 months	Acitretin, topical calcipotriol/betamethasone	Near-complete clearance of papules and plaques	No AEs
Zhao et al., 2025 [109]	Nail lichen planus	Case report	1 (F, 33 y)	Upadacitinib 15 mg QD PO	6 months	9 months	Corticosteroids, retinoids, topical steroids	NALSI score decreased 146 → 37; mild recurrence after dose reduction	No AEs
Houpe et al., 2025 [111]	PLEVA	Case report	1 (M, 67 y)	Upadacitinib 15 mg QD PO	NR	NR	Topical corticosteroids; topical ruxolitinib; deucravacitinib 6 mg QD; dupilumab 300 mg SC q2w	Resolution of rash with post-inflammatory hyperpigmentation	Breakthrough seizure related to underlying epilepsy requiring hospitalization; upadacitinib discontinued
Cirone & Lovegrove, 2025 [114]	Dupilumab-induced psoriasis in atopic dermatitis	Case report	1 (F, 66 y)	Upadacitinib 15 mg QD PO	≥1 year	≥1 year	Dupilumab; apremilast; oral prednisone; topicals	Clear skin/excellent response after switch; sustained >1 year	No AEs
Wang et al., 2023 [115]	Nail psoriasis	Case report	1 (F, 13 y)	Upadacitinib 15 mg QD PO	5 months	24 weeks	Oral Chinese traditional medicine; topical glucocorticoid gels (ineffective)	Near-complete remission by week 20; maintained at week 24	No AEs
Van Eycken et al., 2023 [119]	Therapy-resistant pyoderma gangrenosum	Case report	1 (F, 65 y)	Upadacitinib 15 mg QD PO	24 weeks	24 weeks	Prednisone, cyclosporine, infliximab, etanercept, secukinumab, apremilast	Complete remission after 12 weeks, sustained at 24 weeks	No AEs
Ge et al., 2025 [120]	Facial pyoderma gangrenosum	Case report	1 (M, 20 y)	Upadacitinib 15 mg QD	NR	NR	Steroids; cyclosporine	Complete healing	No AEs
Duque-Clavijo et al., 2025 [121]	Sweet syndrome	Case report	1 (NR)	Upadacitinib (dose NR) PO	NR	NR	NR	Successful treatment (per title)	No AEs
Lei & Zhao, 2025 [122]	Papuloerythroderma of Ofuji	Case report	1 (NR)	Upadacitinib (dose NR) PO	NR	NR	NR	Successful treatment (per title)	No AEs
Zheng et al., 2024 [123]	Generalized eosinophilic pustular folliculitis	Case report	1 (NR)	Upadacitinib (dose NR) PO	NR	NR	NR	Improvement reported with JAK inhibitor (per title)	No AEs
Islam et al., 2024 [125]	Recalcitrant dissecting cellulitis of the scalp	Case report	1 (M, 26 y)	Upadacitinib 15 mg BID + topical antimicrobials + oral antibiotics + corticosteroid injections	2 months	2 months	Topicals, antibiotics, corticosteroids, intralesional steroids	Marked improvement in pain, drainage, bleeding; improved quality of life	No AEs
Song et al., 2023 [129]	Refractory pityriasis rubra pilaris (type I adult-onset)	Case report	1 (F, 81 y)	Upadacitinib 15 mg QD PO	4 weeks	Ongoing	Acitretin, corticosteroids, dupilumab, ixekizumab	Nearly complete clearance within 4 weeks; hair regrowth; normalized LFTs	No AEs
Saad et al., 2023 [130]	Refractory pityriasis rubra pilaris	Case report	1 (F, 26 y)	Upadacitinib 15 mg → 30 mg QD PO	6 weeks	6 weeks	Ustekinumab, ixekizumab, isotretinoin, phototherapy, topicals	~65% improvement in BSA at 6 weeks	Mild headache
Hu et al., 2023 [133]	Discoid lupus erythematosus	Case report	1 (F, 26 y)	Upadacitinib 15 mg QD PO (monotherapy) + entecavir 0.5 mg QD	28 weeks	28 weeks	Hydroxychloroquine; prednisone; doxycycline/isotretinoin	Rapid and sustained improvement; RCLASI 5 → 1; relapse on alternate-day dosing, remission restored with daily dose	Mild acne (managed with benzoyl peroxide); normal labs; HBV viral load decreased
Huang et al., 2024 [135]	Anti-MDA5-positive amyopathic dermatomyositis	Case report	1 (F, 35 y)	Upadacitinib 30 mg QD PO + hydroxychloroquine + tacrolimus ointment	6 weeks	6 months	Methylprednisolone, tripterygium, thalidomide, hydroxychloroquine	Complete remission within 6 weeks; anti-MDA5 Ab titer reduced; maintained remission	No AEs
Maione et al., 2024 [136]	Amyopathic dermatomyositis	Case report	1 (NR)	Upadacitinib (dose NR) PO	NR	NR	NR	Successful treatment (per title)	No AEs
Sohn et al., 2024 [137]	Amyopathic dermatomyositis	Case report	1 (NR)	Upadacitinib (dose NR) PO	NR	NR	NR	Successful treatment after prior JAK inhibitor failure (per title)	No AEs
Sarfaraz et al., 2025 [138]	Diffuse cutaneous systemic sclerosis	Case report	1 (F, 52 y)	Upadacitinib (dose not reported)	NR	NR	Nintedanib, sevelamer, colchicine, tadalafil, steroids, mycophenolate	Improved vascular and cutaneous manifestations	No AEs
Pappa et al., 2024 [139]	Chronic actinic dermatitis	Case report	1 (M, 75 y)	Upadacitinib 15 mg QD PO	≥8 months	8 months	Topical/systemic corticosteroids, calcineurin inhibitors, ciclosporin	Complete clearance within 4 weeks; remained symptom-free at 8 months	No AEs
Morissette & Coulombe, 2025 [141]	Refractory pediatric actinic prurigo	Case report	1 (M, 12 y)	Upadacitinib 15 mg QD PO (patient-tailored/intermittent intake)	NR (ongoing)	NR	Topical potent corticosteroids; topical tacrolimus; polypodium leucotomos; beta-carotene; hydroxychloroquine; methotrexate; mycophenolate mofetil; dupilumab	Rapid improvement in photosensitivity and pruritic lesions; relapse when a dose was omitted with rapid relief after resuming	No AEs
Almaghrabi et al., 2025 [142]	Urticarial vasculitis	Case report	1 (NR)	Upadacitinib (dose NR) PO	NR	NR	NR	Clinical improvement (per title)	No AES
Wang et al., 2025 [143]	Livedoid vasculopathy	Case report	1 (F, 54 y)	Upadacitinib 15 mg QD	NR	NR	Anticoagulants; steroids	Ulcer healing	No AEs
Su et al., 2024 [145]	Bullous pemphigoid coexisting with psoriasis vulgaris	Case report	1 (NR)	Upadacitinib (dose NR) PO	NR	NR	NR	Clinical improvement (per title)	No AEs
Slater et al., 2023 [149]	Generalized granuloma annulare	Case report	1 (F, 57 y)	Upadacitinib 15 mg QD PO	4 weeks	4 weeks	Clobetasol, phototherapy, ROM regimen, intralesional steroids, ruxolitinib cream	Complete clearance in 4 weeks, maintained on therapy	No AEs
Coican et al., 2024 [150]	Generalized granuloma annulare	Case report	1 (NR)	Upadacitinib (dose NR) PO	NR	NR	NR	Successful treatment (per title)	No AEs
Chen et al., 2024 [151]	Generalized papular granuloma annulare	Case report + literature review	1 (NR)	Upadacitinib (dose NR) PO	NR	NR	NR	Effective treatment (per title)	No AEs
Youssef & Bordone, 2023 [156]	Alopecia universalis	Case report	1 (NR)	Upadacitinib (dose NR) PO	NR	NR	NR	Effective treatment (per title)	No AEs
Murphy et al., 2023 [157]	Refractory Hailey–Hailey disease	Case report	1 (F, 65 y)	Upadacitinib (dose not reported)	NR	NR	Triamcinolone, gentamicin, calcipotriene, minocycline, fluconazole, acitretin	Complete clearance of lesions	No AEs
Safadi et al., 2024 [158]	Cutaneous sarcoidosis	Case report	1 (NR)	Upadacitinib (dose NR) PO	NR	NR	NR	Recalcitrant cutaneous sarcoidosis improved (per title)	No AES
Castillo et al., 2022 [159]	Erythrodermic mycosis fungoides	Case report	1 (NR)	Upadacitinib (dose NR) PO	NR	NR	NR	Clinical response reported (per title)	No AEs
Mao et al., 2025 [160]	Atypical tinea corporis with Id reaction	Case report	1 (NR)	Upadacitinib (dose NR) PO	NR	NR	NR	Successful treatment reported (per title)	No AEs
Danese et al., 2025 [161]	Cutaneous pseudolymphoma (IgG4+)	Case report	1 (F, 41 y)	Upadacitinib 30 mg QD PO	6 months	6 months	Topical/systemic steroids; hydroxychloroquine; tacrolimus	Marked reduction in nodules within 4 weeks; continued improvement	No AEs
Zhou et al., 2025 [162]	SJS/TEN overlap	Case report	1 (F, 54 y)	Upadacitinib 15 mg QD + steroids	NR	NR	Systemic steroids	Rapid improvement	No AEs
Deutsch et al., 2023 [163]	Persistent erythema multiforme	Case report	1 (F, 56 y)	Upadacitinib 15 mg QD PO + prednisone 15 mg daily	9 weeks	5 months	Corticosteroids, apremilast, mycophenolate mofetil, IVIG	Near-complete skin clearance in 9 weeks; sustained response	No AEs
Wachuku et al., 2023 [164]	Polycythemia vera-associated pruritus	Case report	1 (F, 40 y)	Upadacitinib 15 mg QD	NR	NR	Antihistamines; phlebotomy	Complete pruritus resolution	No AEs
Tang L. et al., 2024 [165]	Adult-onset Still’s disease with persistent pruritic lesions	Case report	1 (F, 52 y)	Upadacitinib 15 mg QD PO (after baricitinib 1 mg BID) + methylprednisolone	≥10 months (ongoing)	12 months (JAKi therapy)	High-dose methylprednisolone; methotrexate; NSAIDs; antihistamines; baricitinib	Complete resolution of pruritic eruption and laboratory abnormalities by 1 month; enabled major steroid taper	No AEs

Abbreviations: AA, alopecia areata; AEs, adverse events; ALT, alanine aminotransferase; BID, twice daily; BSA, body surface area; CRP, C-reactive protein; DMARDs, disease-modifying antirheumatic drugs; HBV, hepatitis B virus; HCQ, hydroxychloroquine; HS, hidradenitis suppurativa; IGA, Investigator’s Global Assessment; IHS4, International Hidradenitis Suppurativa Severity Score System; IL-6, interleukin-6; IVIG, intravenous immunoglobulin; JAKi, Janus kinase inhibitor; LFTs, liver function tests; LPP, lichen planopilaris; MMF, mycophenolate mofetil; MTX, methotrexate; NALSI, Nail Lichen Planus Severity Index; NB-UVB, narrowband ultraviolet B; NR, not reported; NSAIDs, nonsteroidal anti-inflammatory drugs; PGA, Physician’s Global Assessment; PLEVA, pityriasis lichenoides et varioliformis acuta; PO, per os; QD, once daily; RCLASI, Revised Cutaneous Lupus Erythematosus Disease Area and Severity Index; ROM, rifampin–ofloxacin–minocycline regimen; SC, subcutaneous; SJS/TEN, Stevens–Johnson syndrome/toxic epidermal necrolysis; TCI, topical calcineurin inhibitor; TCS, topical corticosteroids; UC, ulcerative colitis; VES, Vitiligo Extent Score; WI-NRS, Worst Itch Numeric Rating Scale.

## 6. Results: Ritlecitinib

### 6.1. Current Indications

Ritlecitinib is a selective inhibitor of JAK3 and the TEC kinase family. It is approved by both the FDA and EMA at the dosage of 50 mg daily for the treatment of patients aged 12 years and older with severe alopecia areata [8,9,166,167,168,169].

### 6.2. Off-Label Use

Given its relatively recent approval in 2023, the number of published case reports and case series describing the off-label use of ritlecitinib in dermatology remains limited. To date, the available literature includes two case reports, one involving oral lichen planus [170] and the other vitiligo [171].

A more detailed overview of the data is provided in the table below (Table 4).

#### 6.2.1. Oral Lichen Planus

Ritlecitinib, a selective JAK3/TEC inhibitor, has recently shown promise as a targeted therapeutic option in severe, treatment-refractory erosive oral lichen planus (OLP). In this report, a 60-year-old woman with painful erosions, desquamative gingivitis, and persistent mucosal ulcerations, unresponsive to prolonged courses of prednisone, doxycycline, and dexamethasone oral rinses, experienced rapid symptomatic improvement within 48 h of initiating ritlecitinib 50 mg daily. Attempts to taper ritlecitinib led to prompt disease flare, whereas resumption of daily dosing restored control, enabling the patient to remain steroid-free for the first time in two years. The response was durable and well-tolerated, with no significant laboratory abnormalities throughout treatment. OLP is driven by cytotoxic CD8^+^ T-cell activity and Th1/Th17-skewed inflammation mediated by interferon-γ and downstream JAK–STAT signaling. Importantly, lesional tissue demonstrates upregulated JAK3 expression, and inhibition of this isoform can suppress the T-cell-mediated inflammatory cascade responsible for epithelial damage. The rapid, reproducible improvement observed in this case is therefore consistent with targeted blockade of JAK3-dependent pathways in erosive OLP [170].

#### 6.2.2. Vitiligo

This report highlights that ritlecitinib, a selective JAK3 inhibitor, may be more suitable than JAK-1 selective molecules (namely abrocitinib) in certain presentations of vitiligo. A 67-year-old man with severe atopic dermatitis (AD) and pre-existing vitiligo was started on abrocitinib 100 mg daily, achieving rapid and significant improvement of pruritus and eczema. However, over the first month of treatment, his vitiligo lesions expanded markedly, most prominently on the face, despite continued control of AD, with Wood lamp examination and biopsy confirming active vitiligo progression. Upon discontinuation of abrocitinib and initiation of ritlecitinib 50 mg daily, disease activity stabilized, and repigmentation began within two months, with both Total Vitiligo Area Scoring Index (T-VASI) and Facial Vitiligo Area Scoring Index (F-VASI) scores declining and no recurrence of AD lesions [171].

Vitiligo skin shows concurrent upregulation of JAK1 and JAK3, but the relative contribution of each pathway may vary across patients. The authors propose that selective JAK3 blockade may better suppress the IFN-γ–CXCL10 axis driving melanocyte destruction, while JAK1 inhibition alone may be insufficient or, in rare cases, may even perturb the local cytokine balance halting depigmentation. This differential responsiveness provides a plausible explanation for the patient’s worsening on abrocitinib and improvement on ritlecitinib [171].

## 7. Discussion

In this review, we analyzed the available literature on the off-label dermatologic use of oral JAK inhibitors, identifying a total of 35 articles on baricitinib (45 patients), 45 articles on abrocitinib (63 patients), 55 articles on upadacitinib (94 patients), and 2 articles on ritlecitinib (2 patients). Collectively, these reports provide an expanding body of real-world evidence supporting the potential role of JAK inhibition across a broad spectrum of dermatologic conditions beyond approved indications, including lichen planus, prurigo nodularis, psoriasis, and granulomatous diseases [172].

Overall, oral JAK inhibitors demonstrated favorable tolerability, with adverse events reported in a minority of cases and predominantly described as mild and transient. Serious adverse events were uncommon, and treatment discontinuation due to toxicity was rare. However, safety findings derived from off-label case reports and small case series should be interpreted cautiously, as adverse events may be under-reported and follow-up is often limited and non-standardized. In contrast, randomized trials and large real-world studies in approved indications provide a more systematic safety characterization. Therefore, the reassuring safety signals observed in off-label reports should be contextualized within this broader evidence base when considering clinical translation and monitoring strategies.

Across the reviewed reports, these agents showed consistent efficacy in a wide range of inflammatory skin diseases.

A key and recurring finding was the rapid onset of clinical response, with many patients experiencing meaningful improvement within days to weeks after treatment initiation. This rapid efficacy was often accompanied by durable disease control, as treatments were frequently continued for several months with sustained clinical stability. Notably, in many cases, dose reduction or treatment discontinuation did not result in immediate relapse, and remission was maintained in a substantial proportion of patients, suggesting a potential disease-modifying effect in selected conditions.

Notwithstanding these encouraging signals, the case-based nature of the available literature entails intrinsic methodological limitations. Case reports and small case series are inherently subject to publication bias, favoring the reporting of positive outcomes, and are characterized by substantial heterogeneity in patient characteristics, disease severity, treatment regimens, outcome measures, and follow-up duration.

Nevertheless, the breadth and consistency of responses across distinct disease categories justify an exhaustive mapping approach, aimed at providing clinicians with a practical reference framework for off-label use.

## 8. Conclusions

Although larger prospective studies and controlled trials are still needed to better define efficacy, safety, and optimal treatment strategies, this review highlights the broad off-label therapeutic potential of oral JAK inhibitors in dermatology. Their rapid clinical effectiveness, frequent maintenance of remission even after dose tapering or discontinuation, and overall favorable tolerability profile underscore their value as promising options for patients with complex or treatment-refractory dermatologic diseases.

These preliminary data underline the need for the development of prospective studies and randomized clinical trials to further explore the role of JAK-inhibitors in different dermatological conditions.

## Figures and Tables

**Table 1:** Summary of published case reports and case series describing off-label dermatologic uses of baricitinib. Clinical indications marked with an asterisk (*) represent conditions for which the drug is currently being investigated in clinical trials. Underlined clinical indications indicate the main off-label condition for which the drug was used. **Bold** was used to highlight case series.

First Author/Year	Indication	Study Design	Number of Patients	Drug/Dose/Formulation/Association	Treatment Duration	Follow-Up	Previous Systemic Treatments	Clinical Outcome	Adverse Events/Safety
Reviron et al., 2024 [53]	Vitiligo *	**Case series**	5 (3 M, 2 F, 39–79 y)	Baricitinib 4 → 2 mg/day + heliotherapy	4–15 months	4–15 months	Topicals, MTX, PUVA	PR–CR—VES ↓ 41%	Mild CK/LDL/TG ↑
Li et al., 2023 [51]	Vitiligo *	**Case series**	2 (F 17 y, F 56 y)	Baricitinib 2 mg BID + NB-UVB	6–8 months	-	Steroids, tacrolimus	CR—>75% repigmentation	No AEs
Dunn et al., 2023 [31]	Frontal Fibrosing Alopecia *	**Case series**	3 (3 F, 42–52 y)	Baricitinib 4 mg/day PO (+topical ruxolitinib)	1–2 months	6 months	Multiple	PR–CR—Resolution of scale/erythema	No AEs
Burleigh et al., 2023 [36]	ISG15 deficiency (interferonopathy)	**Case series**	2 (F 4 y, M 2 y)	Baricitinib 2 mg BID PO	Ongoing	-	Steroids (1 mg/kg/day)	CR	No AEs
Guo et al., 2024 [37]	CANDLE syndrome	**Case series**	2 (NR)	Baricitinib 2 mg TID (child)	3 months → 3 years	3 years	-	CR—Rash resolved, IFN score normalized	BK virus reactivation
Kim & Kang, 2022 [49]	Granuloma annulare	**Case series**	2 (F 67 y, F 40 y)	Baricitinib 4 mg/day PO	1–1.5 months	14 weeks	MTX, CsA, NB-UVB	CR—Lesions cleared	No AEs
Burningham et al., 2022 [19]	Mucous membrane pemphigoid *	Case report	1 (F, 69 y)	Baricitinib 2 mg/day PO + Methotrexate 20 mg/week	27 months	Ongoing	Multiple (Rituximab, IVIG, MTX)	CR—Healing oral/ocular lesions	No AEs
Moussa et al., 2022 [22]	Lichen planus pemphigoides *	Case report	1 (M, 36 y)	Baricitinib 3.4 mg BID → QD	6 months	6 months	Steroids, MTX, CsA	CR—Resolution of lesions	No AEs
Yoshikawa et al., 2020 [24]	Rheumatoid Arthritis + Bullous Pemphigoid/Lupus Erythematosus under biologics *	Case report	1 (F, 65 y)	Baricitinib 4 mg/day	Months	12 months	ADA, TCZ, abatacept	CR—Arthritis control, BP resolved	No AEs
Kreuter et al., 2022 [26]	Subacute Cutaneous Lupus Erythematosus + Frontal Fibrosing Alopecia *	Case report	1 (F, 62 y)	Baricitinib 4 mg/day → 2 mg	2 months + maintenance	6 months	HCQ, MTX, rituximab	CR—SCLE clearance	No AEs
Bechard et al., 2024 [35]	Pyoderma gangrenosum * (refractory)	Case report	1 (F, 78 y)	Baricitinib 4 mg/day PO + Prednisone taper	12 weeks	13 weeks post-withdrawal	Steroids, colchicine, IVIg	PR → relapse after withdrawal	Septic arthritis (unrelated)
Zhou et al., 2023 [40]	Pyoderma gangrenosum * (associated with eczema)	Case report	1 (F, 38 y)	Baricitinib 4 mg/day	Weeks	Ongoing	ADA, secukinumab	CR—Lesions healed	No AEs
Mumford et al., 2020 [52]	Vitiligo * + Rheumatoid Arthritis	Case report	1 (M, 67 y)	Baricitinib 4 mg/day	8 months	8 months	RA therapies (MTX, biologics)	CR—Repigmentation	No AEs
He et al., 2024 [20]	Epidermolysis Bullosa Pruriginosa	Case report	1 (M, 28 y)	Baricitinib 2 mg/day + antihistamines	2 years	2 years	Topicals	PR → CR—Itch relief	No AEs
Heo & Lim, 2025 [21]	Bullous pemphigoid (diabetic)	Case report	1 (M, 75 y)	Baricitinib 4 mg/day → tapered	10 weeks	14 months	Steroids, antibiotics	CR	No AEs
Xiao et al., 2022 [23]	Bullous Pemphigoid + psoriasis	Case report	1 (M, 83 y)	Baricitinib 4 → 2 mg/day	24 weeks	24 weeks	Topicals	CR—BPDAI 64 → 0	No AEs
Zhou W. et al., 2024 [25]	Infantile bullous pemphigoid	Case report	1 (infant, 6 months)	Baricitinib (PO, low dose)	Weeks	6 months	Steroids, IVIG, dupilumab	CR	No AEs
Rossano et al., 2023 [27]	Juvenile SLE (TREX1)	Case report	1 (F, 13 y)	Baricitinib 2 mg TID + MMF	18 months	18 months	Steroids, MMF, Tacrolimus	CR—Skin/renal improvement	No AEs
Zhan et al., 2023 [28]	Blaschko-linear lupus erythematosus	Case report	1 (M, 28 y)	Baricitinib 4 mg/day	8 months	15 months	Topicals	CR—Lesions flattened	No AEs
Zhou S. et al., 2024 [29]	Cutaneous polyarteritis nodosa	Case report	1 (F, 29 y)	Baricitinib 2–4 mg/day	12 months	12 months	Steroids, immunosuppressants	CR—Ulcers healed	No AEs
Zou et al., 2024 [30]	Linear morphea	Case report	1 (F, 4 y)	Baricitinib 2 mg/day	12 months	12 months	-	CR—Skin softened, LoSCAT improved	No AEs
He et al., 2023 [32]	Nail Lichen Planus	Case report	1 (M, 30 y)	Baricitinib 4 mg/day → 2 mg	6 months	12 months	Topical steroids, acitretin	CR—Complete nail clearance	No AEs
Su et al., 2022 [33]	Lichen sclerosus	Case report	1 (M, 2 y)	Baricitinib 2 mg/day	6 months	6 months	Topicals	PR → CR—Repigmentation	No AEs
Wang et al., 2023 [34]	Lichen sclerosus post-vaccine	Case report	1 (F, 57 y)	Baricitinib 2 mg/day	3 months	3 months	Topical steroids	CR—Resolution of sclerosis	No AEs
Nousari et al., 2021 [38]	Sweet syndrome + Rheumatoid Arthritis	Case report	1 (F, 59 y)	Baricitinib 2 mg/day	10 months	10 months	Prednisone, MTX, biologics	CR—Remission of skin/joint	No AEs
Yang et al., 2024 [39]	SAPHO syndrome	Case report	1 (F, 32 y)	Baricitinib 2 mg BID → 1 mg BID	3 months	3 months	DMARDs, biologics	CR—Pain ↓ 9 → 2	No AEs
Liu et al., 2025 [41]	Eosinophilic pustular folliculitis	Case report	1 (M, 29 y)	Baricitinib 4 mg/day → 2 mg	1 month	2 months	Indomethacin, CsA	CR—Rash resolved	No AEs
Yu et al., 2023 [42]	Dissecting Cellulitis of the Scalp	Case report	1 (M, 15 y)	Baricitinib 4 mg/day + ADA 40 mg q20d	9 months	9 months	Antibiotics, isotretinoin	CR—Lesions resolved	↑ Triglycerides
Aryal et al., 2024 [43]	Eruptive Pruritic Papular Porokeratosis post-COVID-19	Case report	1 (M, 71 y)	Baricitinib 2 mg/day PO + Acitretin 10 mg/day PO	2 weeks + 30 days	1 month	None	CR—Lesion regression, VAS 8 → 2, 12-PSS 45 → 10, DLQI 24 → 5	No AEs
Buttgereit et al., 2021 [44]	Chronic pruritus (refractory)	Case report	1 (F, 71 y)	Baricitinib 2 mg/day PO	2 weeks	3 months	Multiple (dupilumab, UV, antihistamines)	CR—Itch relief (NRS 10 → 0)	No AEs
Gil-Lianes et al., 2024 [45]	Actinic prurigo	Case report	1 (F, 59 y)	Baricitinib 4 mg/day PO	4 months	18 months	HCQ, CsA	CR—Lesions cleared, DLQI 27 → 4	No AEs
Malekan et al., 2024 [46]	Actinic prurigo (pediatric)	Case report	1 (M, 8 y)	Baricitinib 2 mg/day PO	2 weeks	12 weeks	Topicals, antihistamines	CR—Lesions cleared	No AEs
Zhao et al., 2024 [47]	Lipodystrophia centrifugalis abdominalis infantilis	Case report	1 (M, 4 y)	Baricitinib 1 mg/day + HCQ 5.5 mg/kg	10 months	6 months post-stop	Antibiotics, antifungals	CR—Ulcer healed	No AEs
Chen et al., 2024 [48]	Morbihan disease	Case report	1 (F, 37 y)	Baricitinib 2 mg/day PO	4 weeks	-	Hydroxychloroquine, isotretinoin	CR—Reduced erythema/edema	No AEs
Zheng et al., 2024 [50]	Acquired reactive perforating collagenosis	Case report	1 (F, 81 y)	Baricitinib 2 mg/day	8 weeks	3 months	Antihistamines, topicals	CR → relapse post-stop	No AEs

Abbreviations: ADA, adalimumab; AEs, adverse events; BID, twice daily; BK virus, BK polyomavirus; BP, bullous pemphigoid; BPDAI, Bullous Pemphigoid Disease Area Index; CK, creatine kinase; CR, complete response; CsA, ciclosporin; DLQI, Dermatology Life Quality Index; DMARDs, disease-modifying antirheumatic drugs; F, female; HCQ, hydroxychloroquine; IFN, interferon; IVIG, intravenous immunoglobulins; LDL, low-density lipoprotein; LoSCAT, Localized Scleroderma Cutaneous Assessment Tool; M, male; MMF, mycophenolate mofetil; MTX, methotrexate; NB-UVB, narrowband ultraviolet B; NR, Not Reported; NRS, Numeric Rating Scale; PO, per os; PR, partial response; PUVA, psoralen plus ultraviolet A; QD, once daily; RA, rheumatoid arthritis; SAPHO, synovitis, acne, pustulosis, hyperostosis, and osteitis; SCLE, subacute cutaneous lupus erythematosus; SLE, systemic lupus erythematosus; TCZ, tocilizumab; TG, triglycerides; TID, three times daily; TREX1, three prime repair exonuclease 1; UV, ultraviolet; VAS, visual analog scale; VES, Vitiligo Extent Score.

**Table 2:** Summary of published case reports and case series describing off-label dermatologic uses of abrocitinib. Clinical indications marked with an asterisk (*) represent conditions for which the drug is currently being investigated in clinical trials. **Bold** was used to highlight case series.

First Author/Year	Indication	Study Design	Number of Patients	Drug/Dose/Formulation/Association	Treatment Duration	Follow-Up	Previous Systemic Treatments	Clinical Outcome	Adverse Events/Safety
Liang J et al., 2024 [87]	Prurigo nodularis *	**Case series**	2 (1 F 38 y, 1 M 51 y)	Abrocitinib 100 mg/day PO, then tapered in one case	2–4 months	2–4 months	Systemic corticosteroids, hydroxychloroquine, thalidomide, doxycycline, ciclosporin, methotrexate, antihistamines	PR—Marked improvement in pruritus and flattening of nodules	No AEs
Du N et al., 2024 [89]	Chronic spontaneous urticaria	**Case series**	6 (4 F, 2 M; 26–56 y)	Abrocitinib 100 mg/day PO	2–8 months (median 3 months)	2 months–1 year (median 3 months)	Antihistamines, omalizumab, montelukast, hydroxychloroquine, cyclosporine, methotrexate, systemic corticosteroids	CR—5/6 patients, including 2 patients with a 1-year follow-up; PR—1/6 patients	No AEs
Zhang T et al., 2024 [75]	Rosacea	**Case series**	4 (4 F, 35–41 y)	Abrocitinib 100 mg/day PO, then tapered	12–20 weeks (median 16 weeks)	12–20 weeks (median 16 weeks)	Oral antibiotics, hydroxychloroquine, carvedilol, antihistamines	PR—Improvement of pruritus and facial lesions in 2/4 patients; little benefit in the other 2 patients	No AEs
Xu B et al., 2023 [74]	Steroid-induced rosacea	**Case series**	4 (4 F; 35–55 y)	Abrocitinib 100 mg/day PO (in ¾ cases + topical azelaic acid gel and a skin barrier protector)	2–8 weeks (median 6 weeks)	2–8 weeks (median 6 weeks)	Hydroxychloroquine, macrolide antibiotics, systemic corticosteroids	PR—Improvement of erythema and facial vascularity (2/4 cases maintained remission after discontinuation of abrocitinib)	No AEs
De Greef A & Baeck M, 2025 [73]	Solid facial edema (Morbihan syndrome)	**Case series**	3 (3 M, 60–67 y)	Abrocitinib 200 mg/day PO	7.5–9 months (median 9 months)	9 months (median 9 months)	Systemic corticosteroids, isotretinoin, tetracyclines, diuretics, oral metronidazole	PR—Marked reduction in edema/erythema; sustained improvement during treatment	1 transient thrombocytopenia (resolved)
Cai L et al., 2024 [96]	Eosinophilic pustular folliculitis	**Case series**	2 (1 M 50 y, 1 F 40 y)	Abrocitinib 100 mg/day PO	12 weeks -7 months	12 weeks–11 months	Indomethacin, cyclosporine, antihistamines, systemic corticosteroids, diamino diphenyl sulfone	CR—½ patients at 7 months follow-up, after 3 months of therapy discontinuation; PR—½ patients, marked improvement at 11 weeks	No AEs
Bai J et al., 2023 [94]	Lichen amyloidosis	**Case series**	2 (1 F 53 y, 1 M 59 y)	Abrocitinib 100 mg/day PO, then tapered	10 weeks–4 months	10 weeks–4 months	Antihistamines	PR—Significant reduction in pruritus and flattening of lesions	No AEs
Liu X et al., 2024 [69]	Alopecia areata * (associated with atopic dermatitis)	Case report	1 (M, 12 y)	Abrocitinib 200 mg/day PO, reduced to 100 mg/day after 12 weeks	1 year	1 year	None	CR—Full remission of alopecia areata symptoms	No AEs
Liu W et al., 2024 [81]	Granuloma annulare *	Case report	1 (F, 29 y)	Abrocitinib 150 mg/day PO	5 months	5 months	Cyclosporine, hydroxychloroquine, systemic corticosteroids	PR—Patient satisfied with control of her disease, no new lesions	No AEs
Michels A et al., 2024 [82]	Generalized granuloma annulare *	Case report	1 (F, 77 y)	Abrocitinib 200 mg/day PO, then tapered to abrocitinib 100 mg/day, then abrocitinib 100 mg every other day	5 months	11 months	None	CR—Clearance of lesions after 3 months, then no recurrences even after discontinuation	Nausea and herpes labialis during first week of treatment, resolved after switch to abrocitinib 100 mg/day
Sun F et al., 2024 [88]	Prurigo nodularis *	Case report	1 (M, 46 y)	Abrocitinib 100 mg/day PO	2 months	2 months	Systemic corticosteroids, thalidomide	PR—Rapid improvement of pruritus and cutaneous lesions	No AEs
Teng Y et al., 2024 [58]	Mucous membrane pemphigoid	Case report	1 (F, 62 y)	Abrocitinib 100 mg/day PO, tapered	8 weeks	3 months	None; topical corticosteroids, topical tacrolimus	CR—marked pain relief within 3 days, complete resolution by 8 weeks; no relapse during 3-month follow-up	No AEs
Chen P. et al., 2024 [59]	Livedoid vasculopathy	Case report	1 (F; 31 y)	Abrocitinib 100 mg/day PO, tapered	12 weeks	12 weeks	Various systemic treatments	CR—Complete remission after 6 weeks	No AEs
He J et al., 2024 [60]	Nail lichen planus	Case report	1 (F, 39 y)	Abrocitinib 100 mg/day PO, then tapered to 100 mg every other day after 4 months	12 months	12 months	None	PR—Sustained and clinically meaningful improvement, with marked resolution of nail dystrophy.	No AEs
DeBiasio C et al., 2025 [62]	Vulvar lichen planus	Case report	1 (F, 50 y)	Abrocitinib 200 mg/day PO associated with acitretin, apremilast and topical CS and tacrolimus	20 months	20 months	Cyclosporine, prednisone, methotrexate, alitretinoin, mycophenolate	PR—Marked improvement in quality of life; almost lesion-free.	No AEs
Luo Y et al., 2025 [61]	Severe nail lichen planus	Case report	1 (F, 31 y)	Abrocitinib 100 mg/day PO	7 months	7 months	None	PR—Significant improvement in all nails.	No AEs
Xiong X et al., 2024 [63]	Plasma cell balanitis associated with genital lichen sclerosus	Case report	1 (M, 50 y)	Abrocitinib 100 mg/day PO	6 months	6 months	None, circumcision	CR—Improvement after few days and then complete resolution at 1 month.	No AEs
Chen P. et al., 2024 [59]	Hidradenitis suppurativa	Case report	1 (M; 17 y)	Abrocitinib 100 mg/day PO + Doxycycline 100 mg BID (discontinued after 2 weeks); abrocitinib than tapered	10 weeks	10 weeks	Glucocorticoids, doxycycline, retinoic acid, cyclosporine, methotrexate	CR—Near-complete clearance after 6 weeks	No AEs
Chen P. et al., 2024 [59]	Pyoderma gangrenosum	Case report	1 (M, 16 y)	Abrocitinib 100 mg/day PO + Cyclosporine 50 mg BID (discontinued after 4 weeks)	16 weeks	16 weeks	Doxycycline, isotretinoin, corticosteroids, cyclosporine	PR—Rapid improvement within 1 week and near-complete resolution at 4 weeks	No AEs
Estrella MME et al., 2025 [64]	Pyoderma gangrenosum	Case report	1 (F, 54 y)	Abrocitinib 200 mg/day PO	4 months	4 months	Clindamycin and levofloxacine	CR—Ulcer completely healed into a solitary pinkish to skin-colored linear scar	No AEs
Lin Z et al., 2024 [66]	SAPHO syndrome	Case report	1 (M, 17 y)	Abrocitinib 100 mg/day PO + minocycline 50 mg BID for 8 weeks; abrocitinib then tapered	15 months	21 months	Corticosteroids	CR—Complete remission of joint pain, MRI abnormalities and facial skin lesions	No AEs
Yang Z et al., 2025 * [65]	Localized type of GPP	Case report	1 (F, 48 y)	Abrocitinib 100 mg/BID PO, then tapered to abrocitinib 100 mg/day	1 month	1 month	Systemic corticosteroids, acitretin	PR—Resolution of pustules and scales, improvement of erythema	No AEs
Jin S et al., 2024 [70]	Dissecting cellulitis of the scalp	Case report	1 (F, 26 y)	Abrocitinib 100 mg/day PO, then tapered	1 year	1 year	Isotretinoin, antibiotics, corticosteroids, adalimumab	PR—Significant reduction in inflammatory lesions and regression of the nodules	No AEs
Zhang J et al., 2023 [67]	Alopecia universalis secondary to DRESS	Case report	1 (F, 30 y)	Abrocitinib 100 mg/day PO × 2 months → 200 mg/day PO × 2 months, then reduced to 100 mg/day	6 months	6 months	Corticosteroids, tofacitinib 5 mg/day	PR—Regrowth of terminal scalp hair	No AEs
Zhao J et al., 2022 [68]	Alopecia universalis (associated with atopic dermatitis)	Case report	1 (F, 14 y)	Abrocitinib 200 mg/day PO	2 years	2 years	None	CR—Hair regrowth on scalp, eyebrows, limbs, and axillae maintained for 2 years	No AEs
Jin X et al., 2024 [71]	Chronic actinic dermatitis	Case report	1 (M, 70 y)	Abrocitinib 100 mg/day PO, then tapered	6 weeks	6 weeks	Antihistamines, hydroxychloro- quine	PR—Clearance of hypertrophic lesions and relief of pruritus	No AEs
Teng Y et al., 2023 [72]	Perioral dermatitis	Case report	1 (F, 26 y)	Abrocitinib 100 mg/day PO	12 weeks	12 weeks	Tetracycline antibiotics, hydroxychloro- quine	CR—Pruritus improvement after 1 day; complete resolution of lesions after 2 weeks	No AEs
Mao L et al., 2025 [77]	Rosacea aggravated by IPL	Case report	1 (F, 29 y)	Abrocitinib 200 mg/day PO + systemic corticosteroids, both tapered	5 months	5 months	Oral minocycline, systemic corticosteroids	PR—Improvement after 2 weeks and maintained at latest follow-up	No AEs
Ren M et al., 2023 [76]	Granulomatous rosacea	Case report	1 (F, 53 y)	Abrocitinib 100 mg/day PO, then tapered	26 weeks	26 weeks	None	PR—Significant improvement in erythema, swelling and capillary dilatation	No AEs
Fu J et al., 2025 [78]	FBG after mesotherapy	Case report	1 (F, 38 y)	Abrocitinib 100 mg/day PO + prednisone 30 mg/day PO, prednisone tapered	9 weeks	13 weeks	Systemic corticosteroids	CR—Resolution of cutaneous nodules, even after treatment discontinuation	No AEs
Geng Q et al., 2025 [80]	Tattoo-related cutaneous sarcoidosis	Case report	1 (F, 55 y)	Abrocitinib 100 mg/day PO + prednisone 10 mg/day PO	3 months	3 months	Systemic corticosteroids, azathioprine	PR—Marked regression od cutaneous lesions	No AEs
Li Z et al., 2025 [79]	Delayed-onset filler granuloma	Case report	1 (F, 32 y)	Abrocitinib 100 mg/day PO + methylprednisolone 16 mg/day, then tapered	4 weeks	2 months	Systemic corticosteroids	CR—Complete resolution of facial lesion, no recurrences after treatment discontinuation	No AEs
Bizimungu S et al., 2025 [83]	Pseudorheumatoid nodules	Case report	1 (M, 49 y)	Abrocitinib 200 mg/day PO	6 months	6 months	None	PR—Near-complete resolution of lesions; stable disease	No AEs
Satkunanathan S et al., 2024 [84]	Vitiligo	Case report	1 (M, 61 y)	Abrocitinib 100 mg/day PO	2 months	4 months	Systemic corticosteroids, ginkgo biloba	PR—Significant repigmentation after 2 months; maintained improvement 4 months post-treatment (switched to topical tacrolimus)	No AEs
Shao X et al., 2024 [85]	Vitiligo (associated with AD)	Case report	1 (M, 65 y)	Abrocitinib 100 mg/day PO	4 months	4 months	Systemic corticosteroids	PR—Partial repigmentation of vitiligo lesions on face and body	No AEs
Wang Z et al., 2025 [86]	Vitiligo (associated with AD)	Case report	1 (M, 37 y)	Abrocitinib 100 mg/day PO + 308 nm excimer laser therapy	3 months	3 months	Tofacitinib 11 mg/day, upadacitinib 15 mg/day	PR—Approximately 90% repigmentation of facial lesions	No AEs
Bianco M et al., 2024 [92]	Darier disease	Case report	1 (M, 34 y)	Abrocitinib 100 mg/day PO	4 months	4 months	Systemic corticosteroids, cyclosporine	CR—Almost complete clearance of Darier disease lesions and comorbid AD at 4 months	No AEs
Gunyon M et al., 2025 [90]	Hailey-Hailey disease	Case report	1 (F, 60 y)	Abrocitinib 100 mg/day PO	4 months	4 months	Methotrexate, dapsone, acitretin, naltrexone	CR—Complete resolution of lesions after 2 weeks, maintained	Mild, tolerable nausea, constipation
Tang JT et al., 2025 [91]	Netherton syndrome	Case report	1 (F, 29 y)	Abrocitinib	6 months	6 months	Systemic corticosteroids, antihistamines, omalizumab	PR—Marked improvements in symptoms	No AEs
Ye H et al., 2024 [93]	Darier disease	Case report	1 (F, 32 y)	Abrocitinib 100 mg/day PO + acitretin 20 mg/BID PO, both then tapered	11 weeks	11 weeks	Not specified	PR—Marked improvements in symptoms at last follow-up, with tapering of therapy	No AEs
Zhang Y et al., 2024 [95]	Lichen amyloidosis (with comorbid AD)	Case report	1 (M, 32 y)	Abrocitinib 100 mg/day PO	3 months	3 months	None	CR—Marked and rapid improvement of pruritus, lesions flattened	No AEs
Liu B et al., 2025 [97]	Reactive perforating collagenosis	Case report	1 (M, 54 y)	Abrocitinib 100 mg/day PO	3 months	3 months	Antihistamines, systemic immunosuppressants (not specified)	PR—Marked improvement at latest follow-up	No AEs
Wu H et al., 2024 [98]	Pityriasis rosea	Case report	1 (F, 25 y)	Abrocitinib 100 mg/day PO	2 weeks	2 weeks	Antihistamines	CR—Improvement after 1 day and resolution at 2 weeks	No AEs
Xia J et al., 2023 [99]	Eruptive pruritic papular porokeratosis	Case report	1 (M, 75 y)	Abrocitinib 100 mg/day PO	1 month	1 month	Antihistamines, systemic corticosteroids, cyclosporine	CR—Resolution of pruritus and cutaneous lesions	No AEs

Abbreviations: AD, atopic dermatitis; AEs, adverse events; BID, twice daily; CR, complete response; CS, corticosteroids; DRESS, drug reaction with eosinophilia and systemic symptoms; FBG, foreign body granuloma; F, female; GPP, generalized pustular psoriasis; IPL, intense pulsed light; M, male; MRI, magnetic resonance imaging; PO, per os; PR, partial response.

**Table 4:** Summary of published case reports and case series describing off-label dermatologic uses of ritlecitinib. Clinical indications marked with an asterisk (*) represent conditions for which the drug is currently being investigated in clinical trials. Underlined clinical indications indicate the main off-label condition for which the drug was used.

First Author/Year	Indication	Study Design	Number of Patients	Drug/Dose/Formulation/Association	Treatment Duration	Follow-Up	Previous Systemic Treatments	Clinical Outcome	Adverse Events/Safety
Katz A et al., 2025 [170]	Oral Lichen Planus	Case report	1 (F, 60 y)	Ritlecitinib 50 mg/day PO + prednisone 2.5 mg/day and doxycyline 100 mg BID; prednisone than suspended and ritlecitinib tapered	3 months	3 months	Corticosteroids, doxycycline	PR—Marked improvement after 48 h and maintained control of the disease after corticosteroids stop	No AEs
Tong Z et al., 2024 [171]	Vitiligo * associated with AD	Case report	1 (M, 67 y)	Ritlecitinib 50 mg/day PO	2 months	2 months	Abrocitinib 100 mg/day	PR- Initial repigmentation of face and trunk patches. No new lesions	No AEs

Abbreviations: AD, atopic dermatitis; AEs, adverse events; BID, twice daily; F, female; M, male; PO, per os; PR, partial response.

## Data Availability

Not applicable as no new data were generated.
